# Tumor cell membrane‐based vaccines: A potential boost for cancer immunotherapy

**DOI:** 10.1002/EXP.20230171

**Published:** 2024-03-28

**Authors:** Muyang Yang, Jie Zhou, Liseng Lu, Deqiang Deng, Jing Huang, Zijian Tang, Xiujuan Shi, Pui‐Chi Lo, Jonathan F. Lovell, Yongfa Zheng, Honglin Jin

**Affiliations:** ^1^ College of Biomedicine and Health and College of Life Science and Technology Huazhong Agricultural University Wuhan China; ^2^ State Key Laboratory of Respiratory Disease, National Clinical Research Center for Respiratory Disease, Guangzhou Institute of Respiratory Health The First Affiliated Hospital of Guangzhou Medical University Guangzhou China; ^3^ Department of Biomedical Sciences City University of Hong Kong Kowloon Hong Kong China; ^4^ Department of Biomedical Engineering University at Buffalo State University of New York Buffalo New York USA; ^5^ Department of Oncology Renmin Hospital of Wuhan University Wuhan China

**Keywords:** cancer immunotherapy, cancer vaccine, tumor cell membrane, tumor‐derived extracellular vesicles

## Abstract

Because therapeutic cancer vaccines can, in theory, eliminate tumor cells specifically with relatively low toxicity, they have long been considered for application in repressing cancer progression. Traditional cancer vaccines containing a single or a few discrete tumor epitopes have failed in the clinic, possibly due to challenges in epitope selection, target downregulation, cancer cell heterogeneity, tumor microenvironment immunosuppression, or a lack of vaccine immunogenicity. Whole cancer cell or cancer membrane vaccines, which provide a rich source of antigens, are emerging as viable alternatives. Autologous and allogenic cellular cancer vaccines have been evaluated as clinical treatments. Tumor cell membranes (TCMs) are an intriguing antigen source, as they provide membrane‐accessible targets and, at the same time, serve as integrated carriers of vaccine adjuvants and other therapeutic agents. This review provides a summary of the properties and technologies for TCM cancer vaccines. Characteristics, categories, mechanisms, and preparation methods are discussed, as are the demonstrable additional benefits derived from combining TCM vaccines with chemotherapy, sonodynamic therapy, phototherapy, and oncolytic viruses. Further research in chemistry, biomedicine, cancer immunology, and bioinformatics to address current drawbacks could facilitate the clinical adoption of TCM vaccines.

## Graphical abstract

The review provides a comprehensive overview of the development and application of tumor cell membrane (TCM)‐based vaccines for cancer protection and therapy. A detailed description of structure of the TCM‐wrapped particles (TCMPs), modification of TCMPs, membrane hybridization, and related adjuvants is given. As a derivative of TCM, the vaccines for tumor‐derived‐extracellular vesicles (TEVs) are also discussed, which include engineering and approaches to treat tumor cells for TEVs. At last, an outlook based on the perspectives and limitations of TCM vaccines are presented.

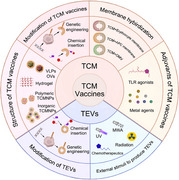

## 1 INTRODUCTION

To initiate and maintain anti‐tumor immunity, tumor antigens and proinflammatory signals must be transmitted to the adaptive immune system. In the tumor microenvironment (TME), the absence of germline‐encoded markers and the occurrence of novel tumor cell antigens cause failure in self‐recognition by natural killer (NK) cells, macrophages, and neutrophils, resulting in immune attack.^[^ ^1^, ^2^, ^3^, ^4^ ^]^ Antigen presenting cells (APCs), such as dendritic cells (DCs), capture tumor antigens stimulated by damage‐associated molecular patterns (DAMPs) from dead tumor cells, inducing their maturation.^[^ ^5^ ^]^ After processing and presenting tumor antigens on major histocompatibility complex (MHC) molecules, DCs migrate into secondary lymphoid organs and transfer antigens to lymph node resident DCs, activating naive T helper cells (CD4^+^ T cells) and cytotoxic T cells (CD8^+^ T cells) to kill tumor cells in TMEs through T cell receptor (TCR)‐MHC matching, co‐stimulatory molecules, and proinflammatory cytokines.^[^ ^6^, ^7^, ^8^, ^9^ ^]^

Immunotherapy for cancer has seen progress in clinical practice in the past decade, with several immunological mechanisms unveiled during that time. The development and widespread application of immune checkpoint inhibitors (ICIs) have demonstrated the importance of tumor immune micro‐environment reshaping. However, because of the lack of transmission of tumor information, which results in indiscriminate immune activation, triggering immune cell action against host tissues, ICIs can cause immune‐related adverse events (irADs).^[^ ^10^ ^]^ In addition to ICIs, the adoptive transfer of modified immunocytes, such as DCs and chimeric antigen receptor (CAR) T and NK cells, can trigger a precise tumor‐specific immune response by packing tumor antigens and proinflammatory signals, which have been proven to effectively inhibit the progression of many tumors.^[^ ^11^, ^12^, ^13^ ^]^ However, these cell‐based therapies face challenges in cancer therapies, including off‐target toxicity, challenges for target selection, high cost of manufacture, and limited infiltration into solid tumors.^[^ ^14^ ^]^

Cancer vaccines containing selected tumor antigens and adjuvants are widely used therapeutic manipulations for the stimulation of tumor‐specific lymphocytes via tumor information delivery at relatively low costs. Cohen and Steinman discovered the immune functions of DCs in 1974, explaining Coley's discovery as the outcome of the transmission of tumor antigens from dead tumor cells to T cells through antigen uptake and the presentation of DCs.^[^ ^15^ ^]^ This discovery set the foundation for the development of tumor‐derived antigen‐related tumor vaccines, which became the driving force behind the popularity of tumor‐associated antigens (TAAs) since the 1980s. Human epidermal growth factor receptor‐2 (HER2), melanoma‐associated antigens (MAGE), Mucin 1 (MUC1), New York‐esophageal cancer 1 (NY‐ESO‐1) and human papilloma virus (HPV) E6 and E7 proteins have been identified as TAA or tumor‐specific antigens, encompassing differentiated, overexpressed or external molecules that are effective in inhibiting tumor growth in clinical trials.^[^ ^16^, ^17^, ^18^, ^19^, ^20^, ^21^ ^]^ Cervarix, Gardasil, and Gardasil 9, the licensed HPV vaccines, have been extensively used in HPV prophylaxis, leading to cervical cancer prevention.^[^ ^20^ ^]^ However, some other vaccines targeting particular tumor antigens have failed to significantly prolong the median overall survival (OS) of patients with some cancers owing to difficulties in finding specific molecules or combinations against the tumor antigen diversity in various patients.^[^ ^22^, ^23^ ^]^ In addition to TAA vaccines, other vaccines, such as tumor cell lysate‐ or inactive tumor cell‐made allogenic tumor vaccines, have emerged. However, lysate‐ or inactive tumor cell‐made allogenic tumor vaccines, which can supply an extensive spectrum of antigens, are still under investigation. The GVAX vaccine platform, tested in clinical trials, consists of irradiated allogenic cancer cell lines that overexpress the granulocyte macrophage‐colony stimulating factor (GM‐CSF), which activates APCs and controls cancer progression.^[^ ^24^ ^]^ Prompted by this, several clinical trials for allogenic tumor vaccines derived from tumor cells that secrete GM‐CSF, IL‐2, IL‐7, interferon‐γ (IFN‐γ), CD40, and CD80 have been carried out for prevention and therapeutic effects.^[^ ^25^ ^]^ Most recently, neoantigens have been conspicuous in studies on cancer vaccine individuation. These antigens vary from patient to patient, for they are generated uniquely from continuous gene mutations, virus infections, and post‐translational modifications in each individual.^[^ ^26^ ^]^ Without germ line encoding, neoantigens can scarcely be found in the reservoir of central tolerance, resulting in more vigorous T‐cell responses and significant tumor inhibitory effects.^[^ ^27^ ^]^ The advent of next‐generation sequencing has significantly accelerated the process of new tumor epitope screening, making it viable for the wide application of neoantigen vaccines. Nevertheless, unearthing neoantigens for the assembly of vaccines with ideal therapeutic efficacy remains challenging, for bioinformatics has not been able to completely decipher the nature of vast mutant antigens, including tumor specificity, cell surface expression, and appropriate immunogenicity, which are crucial to effective vaccination.^[^ ^28^ ^]^ Since the unveiling of DC biology, deploying autologous DC vaccines has become an alternative strategy to tumor cells and related antigens in preventing or treating cancer. Sipuleucel‐T, the first DC vaccine approved by the United States Food and Drug Administration (FDA) in 2010, prolongs the overall survival (OS) of patients with prostate cancer.^[^ ^29^ ^]^ However, because of immunosuppressive microenvironments and hurdles in the manufacturing process, no novel DC vaccine has successfully fulfilled therapeutic expectations.^[^ ^30^ ^]^

In recent decades, a considerable number of studies have probed vaccines that encapsulate tumor cell membranes (TCM) as outstanding carriers of a rich supply of antigens that can trigger tumor‐specific immunity. The cell membrane, which is one of the most fundamental cell components, not only acts as a barrier, but also mediates cell adhesion, signal transduction, mass transfer, enzymatic activity, endocytosis, and immunological recognition.^[^ ^31^ ^]^ Along with remarkable biocompatibility, TCMs allow the insertion of multiple extrinsic molecules without structural instability or immune‐mediated clearance while facilitating physical and chemical modifications.^[^ ^32^ ^]^ Furthermore, the existence of various engineering technologies provides options for customizing the components and structures of the membrane, enabling the controllable display and release of functionalized biological macromolecules. Therefore, TCMs can be regarded as a platform that delivers a complex of several tumor antigens and adjuvants with immunostimulatory molecules. This review examines advances in the applications and diverse manufacturing processes of tumor membranes and their derivatives in anti‐tumor vaccines, with an emphasis on combination strategies and functionality modifications. Practical solutions for improvement, as well as current challenges that have future implications for clinical transformation, are also discussed.

## 2 TCM TUMOR VACCINES

**FIGURE 1:**
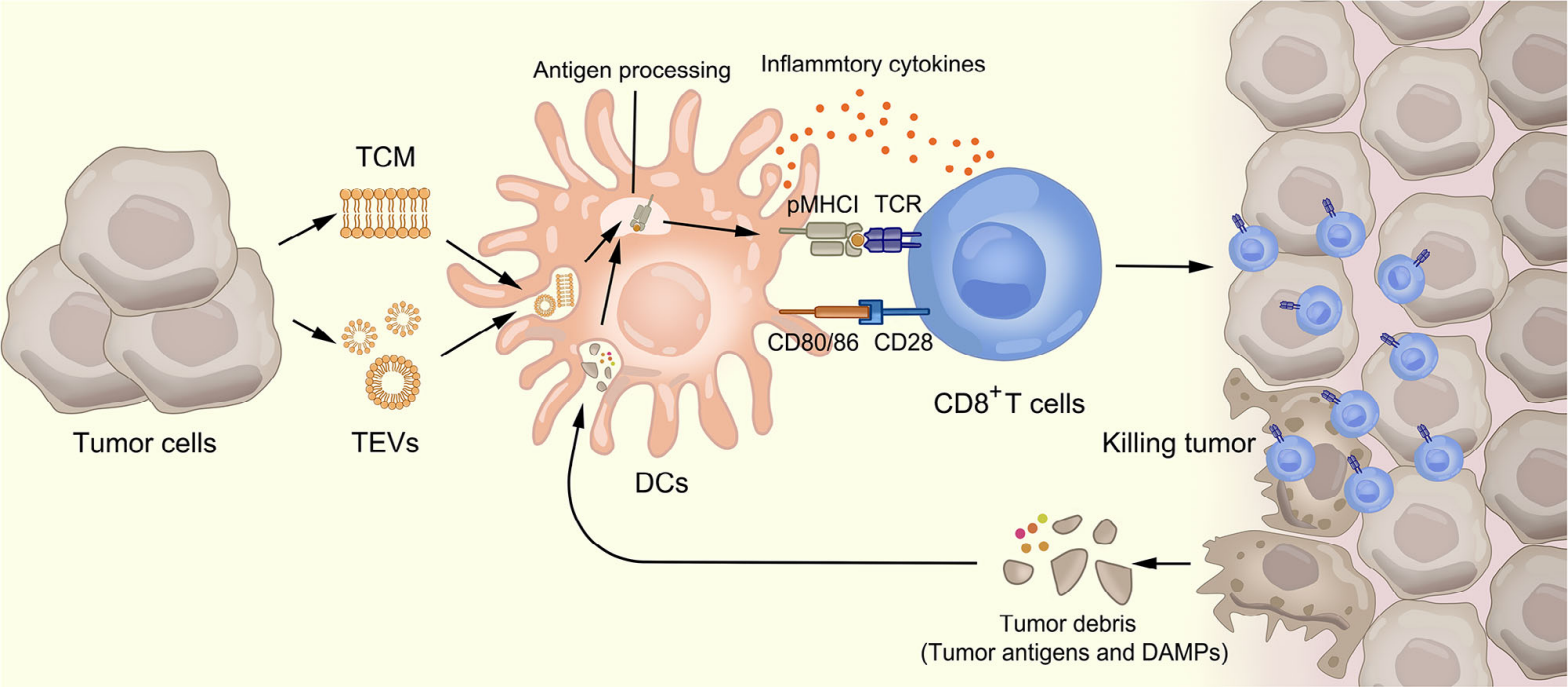
The cascade of tumor elimination via tumor cell membrane (TCM) and tumor‐derived extracellular vesicles. Dendritic cells (DCs) capture tumor information by TCM and its derivation. After antigen processing, DCs present antigen peptide‐loaded major histocompatibility complex (MHC I) complexes on surfaces, activating multiple specific T cells. Cytotoxic T lymphocytes infiltrate in tumor microenvironment and kill tumor cells after recognition of tumor antigens, resulting in the release of tumor debris which is taken by DCs and initiates the next cycle of anti‐tumor immune response.

The TCM, which contains a sizable portion of cancer cell information, is one of the key initiators of anti‐tumor immunity. Generally, the tumor membrane and derivatives with various antigens are taken up by DCs and degraded via proteasomes in the cytosol. After assembling the antigen fragments with the class I MHC α and β2m chains in the endoplasmic reticulum (ER), DCs present peptide‐loaded MHC I complexes on surfaces, activating multiple specific CD8^+^T cells to annihilate tumor cells, resulting in the stimulated release of tumor antigens and DAMPs, which can be captured by DCs.^[^ ^33^, ^34^ ^]^ This tumor killing cascade is constantly augmented until tumor clearance (Figure 1). Multiple specific T‐cell immunity sessions triggered by tumor membrane vaccines reduce the incidence of immune tolerance and escape created by tumor heterogeneity and antigenicity more than vaccines containing a tinge of antigens.

Based on the mechanism above, the requirements of satisfactory vaccines are high efficiency in DC uptake and immune stimulation. To boost efficiency, tumor membranes require distinct processes: nanoparticle construction, modification, and the addition of adjuvants. This part describes in detail the strategies, applications, advantages, and disadvantages of this tumor membrane engineering.

### 2.1 The construction of TCM‐coated nanoparticle (TCMNP) vaccines

**FIGURE 2:**
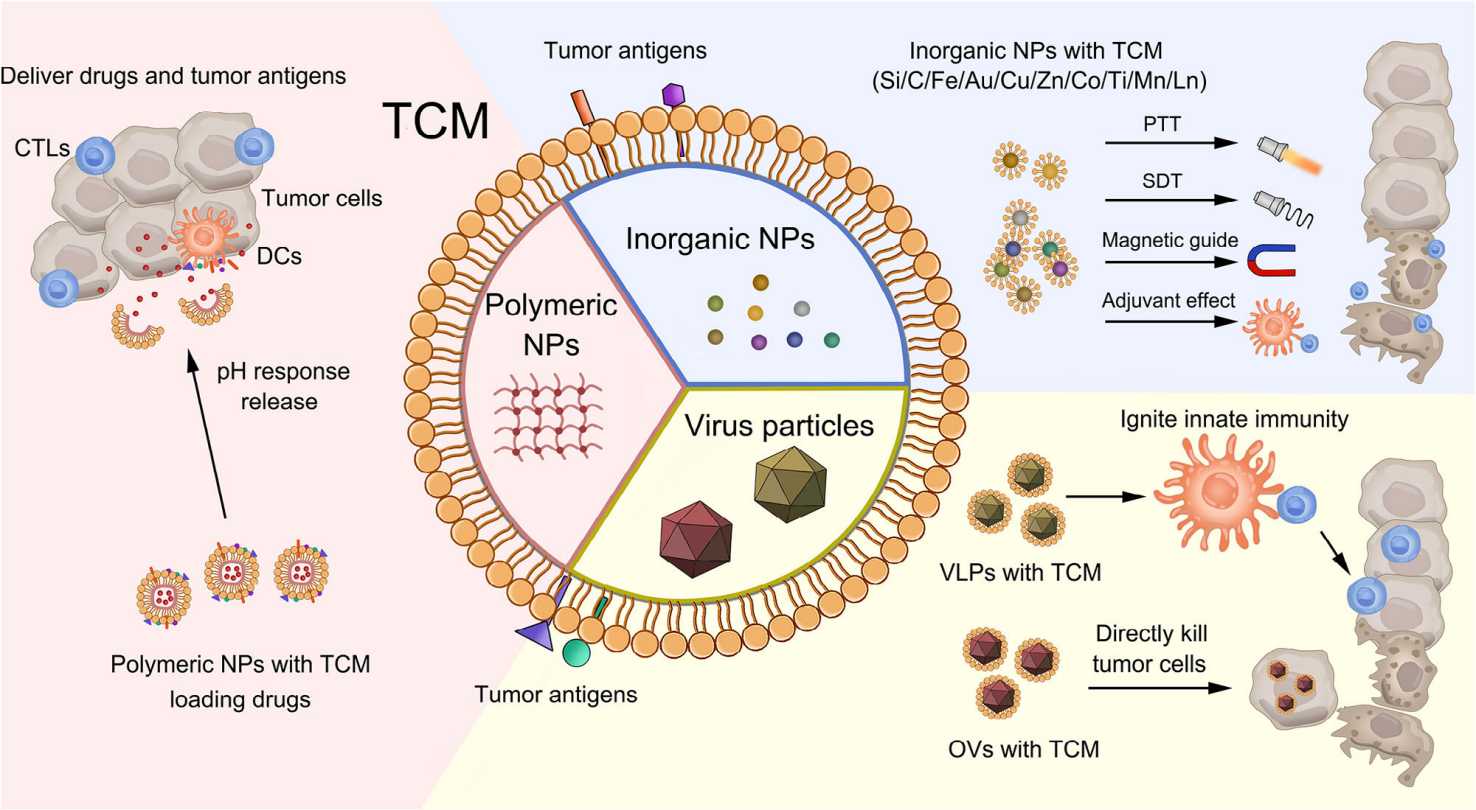
Tumor cell membrane (TCM) wrapped nanoparticle vaccines made by distinct synthetic nanomaterials. The applications and mechanisms of polymeric nanoparticles (NPs), inorganic NPs, and virus particles with TCM were shown.

Due to insufficient immunogenicity, serum instability, and short half‐life, TCMs alone may not induce robust and lasting immune responses. Since the first report on cell membrane‐coated nanoparticles (NPs, ranging from 10–1000 nm),^[^ ^35^ ^]^ attention has focused on nanocarriers loading tumor antigens on the cell surface to treat various cancers thanks to their ability to easily modify and aggregate the cell membrane.^[^ ^36^, ^37^, ^38^, ^39^, ^40^, ^41^ ^]^ NPs and the cell membrane benefit one another mutually. Some physical treatments, like magnetic hyperthermia, photothermal therapy, and photodynamic therapy, can ally with anti‐tumor immunity mediated by different materials in NPs.^[^ ^42^ ^]^ As an associate of NPs, the cell membrane is a natural lipid structure that protects NPs from rejection by the immune system. Furthermore, TCMs facilitate tumor targeting and adhesion through tumor characteristic functional factors such as Galectin‐3, N‐cadherin, E‐cadherin, and the epithelial cell adhesion molecule (EpCAM).^[^ ^43^ ^]^ Reportedly, TCMNPs could treat cancer via effective APCs uptake,^[^ ^44^ ^]^ robust T cell cytotoxicity, and excellent tumor targeting.^[^ ^45^ ^]^ In recent decades, TCMNP platforms have been greatly enhanced with various membrane sources and a wide range of synthetic nanomaterials, for instance, biodegradable polymers, inorganic materials, and virus particles^[^ ^32^, ^46^, ^47^ ^]^ (Figure 2).

#### 2.1.1 Polymeric NPs

With favorable bioavailability, drug delivery, and pharmacokinetics characteristics, polymeric NPs are common inner cores for cell membrane wrapping. They promote phagocytosis in APCs and receptor‐mediated endocytosis, accelerating the uptake of membrane antigens and igniting immune responses.^[^ ^47^ ^]^ Among polymeric NPs, poly (lactic‐co‐glycolic acid) (PLGA) is one of the most common materials for drug delivery because it exhibits controllable biocompatibility and biodegradablility. The double emulsification of PLGA and drugs forms water in‐oil‐in‐water (W/O/W) emulsion, setting the foundation for the core structure of agent encapsulation.^[^ ^48^ ^]^ The size, position of release, and half‐life of PLGA NPs for treatment are critical to treatment, and these properties can be adjusted using specific processing methods.^[^ ^49^ ^]^ In cancer vaccines, TCMs have typically been utilized to wrap PLGA NPs as antigen repertoires. Tumor membrane‐coating PLGA NPs (NP‐R@M‐M) were developed with imiquimod (R837) and mannose modifications, and these NPs accelerated the maturation of DCs with higher levels of IL‐12 and TNF‐α production. NP‐R@M‐M also significantly inhibited the growth of B16F10 tumor cells and synergized immune checkpoint blockades in a mouse model via the anti‐programmed cell death protein 1 antibody (αPD‐1).^[^ ^50^ ^]^ This combination of tumor membranes and PLGA NPs alongside specific APC stimulators has equally been demonstrated to inhibit various tumor types, such as colorectal and breast cancer, effectively.^[^ ^51^, ^52^, ^53^, ^54^ ^]^ Besides acting as antigen providers, cell membranes influence the kinetic characteristics of additional agents in NPs.^[^ ^55^ ^]^ The membranes of some cells, like stem cells and macrophages, have been suggested to decelerate the release of drugs in NPs, resulting in more steady delivery and half‐life prolongation.^[^ ^56^, ^57^ ^]^ Whether TCMs share the same property and approach to adjusting the release position and speed requires further investigation.

#### 2.1.2 Inorganic NPs

With unique physical properties in terms of electrical conductance, optics, and magnetism, inorganic NPs have extensively been applied in cancer vaccines as mediators of diagnosis and physical therapy. The core of inorganic NPs consists of various inorganic materials, such as gold, iron, silica, carbon, titanium, and manganese, which can stimulate innate immunity via the NLRP3 inflamasome, the cGAS‐STING pathway, and reactive oxygen species (ROS).^[^ ^58^, ^59^ ^]^ The organic elements are designed as a shell structure for protecting immune clearance.^[^ ^60^ ^]^ As natural organic materials, TCMs possess most of the properties of artificial organic structures, in addition to tumor antigen provision and tumor targeting assets, pointing to the synergism between TCMs and inorganic cores in cancer therapies. Numerous studies have examined tumor membrane‐wrapping inorganic NPs in combination with tumor vaccines and imaging, photothermal therapy, sonodynamic therapy, and chemotherapy (Table 1).

**TABLE 1:** The application of inorganic NPs in tumor cell membrane vaccines for cancer therapy.

Elements in NPs	Structure of NPs	Combination therapies	Vaccine effect
Fe_3_O_4_	CT26 TCM wrapped Fe_3_O_4_ NPs with chlorin e6	CDT/SDT	Apoptosis of CT26 cells and tumor growth inhibition in mice^[^ ^63^ ^]^
Fe_3_O_4_	TCM encapsulated Fe_3_O_4_@SiO_2_	magnetic guide	Activated NK cells with higher expression of cytotoxic cytokines^[^ ^65^ ^]^
Fe_3_O_4_	PLGA‐loaded Fe_3_O_4_ with Nrf‐2 antagonist coating by TCM	Radiotherapy	Induce ROS and ferroptosis of osteosarcoma^[^ ^67^ ^]^
Au	Au@C‐CCMs	PTT/Immunotherapy	Inhibit HNSCC metastasis by inducing ICD^[^ ^69^ ^]^
Au	Hybrid membrane coated Au@SiO_2_ NPs loading with R837	PTT/Immunotherapy/Starvation therapy	Efficient ICD of breast cancer cells in mice^[^ ^70^ ^]^
Au/Pt	Au@Pt@CM NPs		Oxidation of TMB which augmented ROS‐mediated oxidative damage of breast cancer cells^[^ ^71^ ^]^
CuS	CuS NPs encapsulating sorafenib with TCM	CDT/PTT	94.3% inhibition of HCC growth^[^ ^72^ ^]^
TiO_2_	C‐TiO_2_/TPZ@TCM	CDT/SDT	Apoptosis of CT26 cells and significant tumor growth inhibition^[^ ^73^ ^]^
Fe/Mn/Zn/Co	TCM coated trimagnetic NPs with cell‐penetrating peptide	MFH	Induced intracellular hyperthermia and death of prostate cancer cells^[^ ^76^ ^]^
TiO_2_/MnO_2_	GOx decorated TiO_2_@MnO_2_ core–shell nanoreactor	X‐ray	Augmented ROS‐mediated oxidative damage of B16‐F10^[^ ^161^ ^]^
MnOx	CM@Mn	ICB	Enhanced ROS production, promoted maturation of DCs and macrophage M1 repolarization and synergized ICB^[^ ^162^ ^]^
MnO_2_/Au	MnO_2_ nanosheet coated gold nanorod carrying DOX, and coated with TCM	CDT/PTT	Assisted MRI and inhibited the growth of 4T1 tumor cells^[^ ^163^ ^]^

Abbreviations: CDT, chemodynamic therapy; DOX, doxorubicin; GOx, Glucose oxidase; HCC. hepatocellular carcinoma, HNSCC, neck squamous cell carcinoma; ICB, immune checkpoint blockade; ICD, immunogenic cell death; MFH, magnetic‐fluid‐mediated hyperthermia; MRI, magnetic resonance imaging; NPs, nanoparticles; Nrf‐2, nuclear factor‐erythroid 2‐related factor 2; PLGA, poly (lactic‐*co*‐glycolic acid); PTT, photothermal therapy; ROS, reactive oxygen species; SDT, sonodynamic therapy; TCM, tumor cell membrane; TMB, tetramethylbenzidine; TPZ, tirapazamine.

Fe_3_O_4_, a common metal agent, can combine magnetic resonance imaging (MRI) with photothermal therapy through its magnetic response and pyrogenicity.^[^ ^61^ ^]^ Allied with chemodynamic and sonodynamic therapy, Fe_3_O_4_‐loading tumor membranes have been shown to significantly induce ROS production and suppress CT26 tumor cell growth.^[^ ^62^ ^]^ Moreover, Fe_3_O_4_ nanoclusters have been found to enhance the ability of TCM vaccines to activate DCs, CD8^+^T cells, and NK cells via their superparamagnetism and magnetization, which mediate the magnetic approach to retaining vaccines in TMEs for a long time.^[^ ^63^, ^64^ ^]^ In addition to magnetic targeting, Fe_3_O_4_ NPs taken up by tumor cells are irreversible catalyzers of the Fenton reaction, which produces a large amount of ROS and can promote ferroptosis.^[^ ^65^ ^]^ Yu et al. constructed DHJS (a probe for ROS generation) and Fe_3_O_4_ NPs coating with a hybrid cell membrane of human osteosarcoma and erythrocyte to synergize tumor elimination by ferroptosis induction. These NPs strongly inhibited tumor growth and enhanced the number of infiltrated inflammatory immune cells.^[^ ^66^ ^]^ Gold (Au) NPs are more robust in their photothermal effect than iron owing to their property of localized surface plasmon resonance (LSPR).^[^ ^67^ ^]^ However, the application of Au NP photothermal therapy faces severe challenges, given their inability to target TMEs, which abate tumor toxicity and bring about side effects. Concealing Au NPs with tumor membranes would facilitate their accumulation in tumor sites, as well as provide an antigen pool. As mediators of the immunogenic cell death (ICD) of cancer cells, gold‐carbon or gold‐silica NPs coated with tumor membranes can ignite anti‐tumor immunity and suppress tumor metastasis under near infrared radiation.^[^ ^68^, ^69^ ^]^ Like Fe_3_O_4_, Au NPs also serve as peroxidase‐producing ROS and synergize tumor membrane vaccines.^[^ ^70^ ^]^ In addition to these two metals, copper, zinc, cobalt, titanium, and lanthanide have been proven as vital mediators of physical therapy and deliverers of drug wrappings with tumor membranes to amplify the effectiveness of tumor killing.^[^ ^71^, ^72^, ^73^, ^74^, ^75^ ^]^

#### 2.1.3 Viral particles

Most viral vaccines are made of attenuated or inactivated viruses that can strongly mobilize immunity via viral antigen delivery and TLR activation without infection.^[^ ^76^ ^]^ However, viral particles face numerous challenges in the prevention and treatment of cancer, as they are inclined to trigger anti‐viral immunity instead of a response against tumor antigens. Virus‐like particles (VLPs), which serve as substitutes for completed viruses, comprise viral proteins and other additives without the genetic elements of reproduction and infection, suitably reserving the immunogenicity of original viruses, which makes them popular in viral vaccine preparations.^[^ ^77^ ^]^ Several VLP vaccines against HPV‐induced cancers have been shown to activate robust and sustained humoral immunity with cross‐protection between distinct HPV protein types.^[^ ^78^, ^79^ ^]^ To widen the anti‐carcinoma property, VLPs are assembled as carriers of some tumor‐specific antigens and adjuvants. Nevertheless, natural virus‐derived VLPs can suitably activate adaptive immune responses against viral antigens but are usually inadequate in the anti‐tumor immunity prevention and treatment of non‐viral‐developed cancers. Artificial and biodegradable VLPs, termed “virosomes” can alter this type of activated immunity. Virosomes range from 80–150 nm in diameter and can deliver antigens and adjuvants using their synthetic structures composed of viral phospholipids and glycoproteins.^[^ ^47^ ^]^ In their construction of HER2‐diplaying VLP vaccines to inhibit the growth of breast cancer, Nika et al. used Sf9 insect cell‐overexpressing HIV‐Gag‐HER2 coupling proteins to genetically engineer and deploy VLPs with HER2. Merged with adjuvant AddaVax, the VLPs significantly prolonged survival time and heightened IFN‐γ’s secretion of T cells in mammary carcinoma cell‐burden mice.^[^ ^80^ ^]^ These findings show that the combination of VLPs with tumor antigens is feasible, suggesting that tumor membrane combination with VLPs for tumor vaccination is effective. Integrating antigens from tumor membranes and viruses is possibly the most singular challenge to the application of the hybridization of tumor membranes with VLPs.

As well as VLPs, some tumor‐target viruses can eliminate cancer cells directly instead of only being carriers and stimulators. Oncolytic viruses (OVs), novel and potential cancer therapy associates, selectively reproduce in tumor cells and lyse tumors alongside ICD, overwhelming resistant cancers.^[^ ^81^, ^82^ ^]^ Nevertheless, naked viruses trigger anti‐viral immunity, shortening the circulation of OVs with repeated administration.^[^ ^83^ ^]^ To diminish the immunogenicity of OVs, Fusciello et al. established new OV NPs (ExtraCRAd) using TCM wrappings to preserve OVs from immune clearance as well as enhanced infectivity and oncolytic effects.^[^ ^84^ ^]^ Compared to naked viruses, ExtraCRAd has been observed to suppress A549, B16, and LL/2 subcutaneous tumor progressions with more DC and tumor‐specific CD8^+^T cell infiltrations and lower levels of anti‐virus antibodies in the plasma. One lung cancer model experiment found the survival rate of mice treated with ExtraCRAd to be over 50% 40 days after CMT64 tumor cell inoculation, while mice treated with mock and naked viruses were all dead by then.^[^ ^84^ ^]^ Combining OVs with TCM paves the way for the pluralization of biological engineering and appropriate adjuvant delivery, reinforcing OVs’ targetability and increasing tumor immunocyte infiltration.

### 2.2 Hydrogel

Hydrogels are 3D hydrophilic polymer networks that swell quickly in water and hold a large amount of moisture content owing to their hydrophilic functional groups, like ─OH, ─COOH, ─NH_2_, and ─SO_3_H.^[^ ^85^, ^86^ ^]^ The crosslinked polymer chains in hydrogels create a base for their soft and flexible structures that can be degraded through specific chemical and physical responses depending on the properties of the materials, rendering them appropriate in targeted drug delivery.^[^ ^87^ ^]^ Natural hydrogels, including chitosan, agarose, gelatin, hyaluronic acid, among others, are biocompatibile and biodegradable and are safe for use by the majority of people; however, they may be insufficiently stable to fulfill the requirement of drug delivery.^[^ ^88^ ^]^ Likewise, artificial polymers, such as polyethylene glycol (PEG), polyvinyl alcohol (PVA), and polyacrylamide (PAAM), play key roles in therapies thanks to their designable stability, mechanical strength, and biocompatibility.^[^ ^89^ ^]^ Based on these characteristics, hydrogels are suitable as carriers of cancer vaccines, and this suitability has been demonstrated in several studies.^[^ ^90^ ^]^ The microporous structures of hydrogels promote the recruitment of immunocytes and the expression of inflammatory factors, for example, TLR agonists and manganic salts, which foster the activation of DCs.^[^ ^91^, ^92^ ^]^ In addition, some hydrogel materials themselves can mobilize innate immunity. Chitosan, an agonist of cGAS‐STING, produces natural hydrogels capable of enhancing APC activation. When combined with the TLR9 agonist, CpG, chitosan hydrogels amplify immunocyte infiltration and inflammatory cytokine secretion in TMEs, resulting in the formation of tertiary lymphoid structures (TLSs).^[^ ^93^ ^]^ Sericin/silk fibroin is another example of a natural hydrogel that can activate innate immune cells. Evidence shows that sericin/silk fibroin hydrogel acts robustly alongside TLRs, thereby activating the NF‐κB pathway and instigating M1 macrophage polarization in TMEs.^[^ ^94^ ^]^ Merging TCMs with hydrogels can realize TME target release. Rao et al. designed gelatin hydrogel coated with a tumor membrane degrades in tumor sites in a similar manner to degradation caused by matrix metalloproteinases (MMPs).^[^ ^95^ ^]^ Further inquiries are needed to elucidate how hydrogels assist cancer membranes in targeting tumors and increasing immunogenicity.

### 2.3 Modification of TCM vaccines

Modifying TCM vaccines is necessary to improve the efficiency of tumor targeting and treatment. Conferring novel characteristics to TCM vaccines is possible thanks to their biological and chemical properties. TCM vaccine modification can be achieved in four main ways: (1) the addition of factors promoting anti‐tumor immunity via the genetic engineering of tumor cells from which TCM is derived; (2) the insertion of molecules through chemical methods; (3) the hybridization of TCMs with other cells. These approaches can substantially boost the speed and specificity with which TCM vaccines ignite anti‐tumor immunity.

#### 2.3.1 Genetic engineering

The genetic engineering of tumor cells provides novel pathways for enhancing anti‐tumor immunity. Fundamental to APCs role in tumor elimination, researchers have sought to modify tumor cells as artificial APCs (aAPCs) to create vaccines by presenting tumor antigens, overexpressing makers, and cytokines that induce immune boost.^[^ ^53^, ^96^ ^]^ The co‐stimulatory molecules of B7 family members, including CD80 and CD86, strictly control T cell activation via interaction with CD28 and following the activation of nuclear factor‐κB (NF‐κB) and the mitogen‐activated protein (MAP).^[^ ^97^, ^98^ ^]^ B16‐F10 melanoma cells overexpressing CD80 combined with cell membrane‐coated NPs enable the enhancement of CD25 and CD69 expression, IL‐2 and IFN‐γ secretion, and the proliferation of B16‐F10‐specific cytotoxic T lymphocytes (CTLs).^[^ ^53^ ^]^ In prophylactic and therapeutic treatments in vivo, CD80 overexpressing NPs showed high efficiency in the inhibition of tumor progression and prolongation of survival time.^[^ ^53^ ^]^ CD86, another B7 family member, has also been noted to facilitate cancer repression. Sun Z. et al. found that the genetic addition of CD86 into 4T1 mastadenoma promoted the transformation of “cold tumor” into “hot tumor” via aggregation‐induced‐emission (AIE) photosensitizer loading.^[^ ^96^ ^]^ The insertion of co‐stimulatory molecular agonists is an acceptable alternative to the insertion of B7 members. In Li Y. et al.’s inquiry with genetically engineered tumor cells with the anti‐CD40 single chain variable fragment (scFv), the tumor cell membranes in the vaccine abounded with scFvs, accelerating DC maturation and effectively inhibiting MC38 tumor progression in CD40‐humanized mice.^[^ ^99^ ^]^ Cancer cells can escape from phagocytosis via the connection of CD47 with the signal regulatory protein α (SIRPα, or CD172a) in what is known as a “don't eat me” signal.^[^ ^100^ ^]^ TCMNPs from some cancer cells are rich in CD47, which helps avoid removal by macrophages, prolonging their half‐lives.^[^ ^101^ ^]^

Although progress in genetic engineering has eased the modification of tumor membrane vaccines to boost anti‐tumor immunity, a lot must still be done to mitigate the challenges faced. Viral carriers, like adenoviruses and lentiviruses, exhibit high transfection efficiency in gene addition, knockout, or replacement. Nevertheless, randomly integrating viruses could result in the mutation and destruction of the host genome, causing alterations in host cell characteristics that are unexpected.^[^ ^102^ ^]^ Moreover, off‐target editing occurs regularly after viral transfection owing to the generation of nuclease overexpression by viruses.^[^ ^103^ ^]^ For better biosafety, nonviral vectors are applied in genetic engineering. In contrast, the main mediators for gene delivery, such as liposomes and nanocarriers, are latently toxic, with lower efficiency in cellular uptake and shorter half‐lives.^[^ ^104^ ^]^ Breakthroughs in molecular biology and chemistry have enabled improved transfection efficiency and reduced toxicity through inorganic, polymeric nanocarriers and extracellular vesicles.^[^ ^105^ ^]^ Furthermore, improved and proven electroporation accelerates alien gene entrance and specific site insertion via combination with novel biological methods such as mRNA mediated gene modification.^[^ ^106^, ^107^, ^108^ ^]^ mRNA is considered as an effective way to change the expression spectrum of cells via translating target proteins after being captured by cells with a low risk of gene integration. Genetic engineering by mRNA gradually attracts attentions in biopharmaceuticals since the success of COVID‐19 mRNA vaccines. The safety and effectiveness of mRNA‐mediated genetic engineering of TCM need further evaluation and optimization for a longer half‐life and lower toxicity.

#### 2.3.2 Chemical insertion

The phospholipids and residues of proteins simplify the modification of cell membranes and the lipid outer layers of exosomes. Chemically modifying cell membranes smooths the addition of external molecules at relatively accurate ratios, as well as with longer half‐lives and more effective distribution.^[^ ^109^ ^]^ In inserted molecules, chemical groups, for example, amines, are vital for coupling with membranes. N‐hydroxysuccinimide (NHS) and 1‐ethyl‐3‐(3‐dimethylaminopropyl) carbodiimide (EDC), the most common condensation agents, function as mediators to bind the carboxyl and amine groups to generate amide bonds. Click chemistry is also used in binding via the copper‐catalyzed reaction of azides and alkynes.^[^ ^110^ ^]^ 1,2‐distearoyl‐sn‐glycero‐3‐phosphoethanolamine‐N‐[amino(polyethylene glycol)] (DSPE‐PEG) is a common linker of cell membranes to inserted molecules, such as mannose, aptamers, peptides, and other functional molecules.^[^ ^111^ ^]^ DSPE‐PEG has been proven to successfully bind mannose, TGFβRII neutralizing antibodies with TCMs to promote DC uptake and activation.^[^ ^50^, ^112^ ^]^ In addition to non‐natural molecules, saccharides and lipids can serve as linkers of cell membranes to additives, the combination of which regulates the phenotypes and functions of cells via the regulation of natural biosynthetic pathways.^[^ ^113^ ^]^ Metabolic engineering approaches, including mainly glycoengineering and lipid‐engineering, have been reported to bind pMHC‐I and anti‐CD28 to NPs, which can camouflage as APCs to stimulate CD8^+^T cells.^[^ ^114^ ^]^ The addition of metabolic factors can alter the natural biosynthetic pathways of target cells, impacting the functions of immune cells, like DC activation, macrophage polarization, and CTL cytotoxicity.^[^ ^115^, ^116^, ^117^ ^]^ Biotin‐(Strept) avidin (BA) system is another way for coupling that has been widely used for over half a century. The interaction of biotin and avidin has been considered one of the most specific and stable linking in non‐covalent interaction that the dissociation constant is thousands to millions times higher than the interaction of antibody and antigen.^[^ ^118^ ^]^ Furthermore, biotin shows self‐adjuvant properties that induce the production of IL‐1β, IL‐18, and IL‐12 of APCs, leading to the promotion of Th1‐type helper T cell differentiation when assembled in NPs.^[^ ^119^ ^]^ Therefore, the BA system is suitable for the modification of nano‐vaccine.^[^ ^120^, ^121^ ^]^ Because the complexity of molecule distribution of TCM, application of BA system in TCM vaccines is limited. How to avoid the neutralization of TCM molecules by BA system and increase the abundance of BA needs further investigation.

Another alternative to membrane insertion is membrane‐anchor molecules, for instance, glycosyl phosphatidylinositol (GPI), whose elements are assembled on the ER membrane. Immature GPI‐anchored proteins (GPI‐APs) are linked to GPI through an amide bond generation between the C‐terminus of GPI‐APs and the terminal ethanolamine phosphate of GPI. After modification in the Golgi apparatus and transport by vesicles, GPI‐APs are deposited on the cell membrane.^[^ ^122^ ^]^ As a natural protein attached to membranes via a post‐translational modification, GPI can be designed genetically to combine target inflammatory surface markers and cytokines with excellent biocompatibility.^[^ ^123^ ^]^ For example, one investigation reconstructed CD80 and IL‐12 into GPI‐APs for tumor vaccines by attaching them to the CD59 GPI‐signal sequence,^[^ ^124^ ^]^ and tumor membrane vesicles armed with these GPI‐CD80 and GPI‐IL‐12 demonstrably amplified tumor‐specific T cell immunity and the effectiveness of ICIs in models of triple‐negative breast cancer (TNBC) and head and neck squamous cell carcinoma (HNSCC).^[^ ^125^, ^126^ ^]^ In summary, chemical surface engineering is critical to building additional proteins and membranes; however, the material limitations and toxicity of these proteins and membranes are challenges that must still be overcome.

### 2.4 Membrane hybridization

**FIGURE 3:**
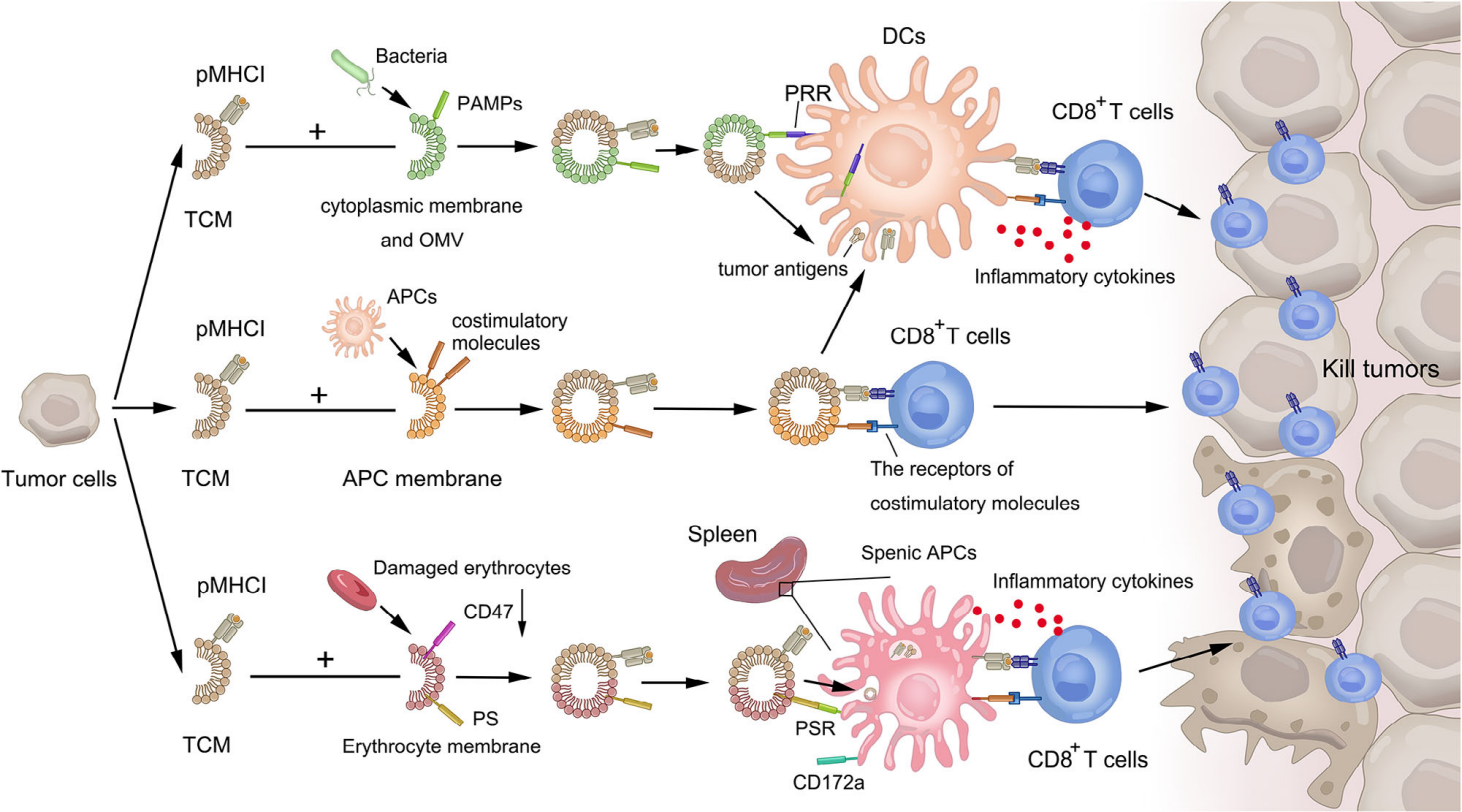
The hybridization of tumor and other cell membrane for tumor vaccines. Tumor and bacterial hybridization is potential to serve as tumor vaccines, for bacterial membrane and outer membrane vesiclesare abundant with pathogen associated molecular patterns (PAMPs) which can trigger innate immunity via the recognition of pattern recognition receptors (PRRs). Antigen‐presenting cells (APCs) express costimulatory molecules that activate CD8+T cells. The combination of tumor and APC membrane functions as artificial APCs while transmit tumor antigens to APCs. Besides, damaged red blood cells highly express phosphatidylserine (PS) which can be recognized by receptors on splenic APCs, and the level of “don't eat me” signal CD47 downregulate, that makes the hybrid membrane of tumor and red blood cells can target and be taken by APCs in spleen.

The “all in one” strategy is applicable in the synthesis of multifunctional materials in the field of nanoparticles through assembling units with diverse functions, such as targeting, long circulation, immune activation, and drug loading.^[^ ^127^ ^]^ Similarly, due to the existence of various functional receptors on the surfaces of different cells, the concept of hybrid cell membranes was proposed to uncover further biomedical applications. Over the past few years, different combinations have emerged within TCMs. Red blood cells, immune cells, and prokaryotic cells have been deployed as combiners via immune activation, targeting, immune escape, and adherence to tumor cells to enhance therapeutic outcomes^[^ ^128^ ^]^ (Figure 3).

Bacterial components, for example, cytoplasmic membranes and outer membrane vesicles (OMVs), yield PAMPs, which can interact with pattern recognition receptors (PRRs) distributed across the surfaces of endothelial cells and immune cells, resulting in the triggering of innate immune responses and adaptive immune promotion.^[^ ^129^, ^130^, ^131^ ^]^ Compared to complete bacterial elements, the hybridization of cells with bacterial membranes reduces the risk of cytokine storms and sepsis without attenuating immunity activation.^[^ ^129^ ^]^ Chen et al. developed a personalized tumor vaccine formulation via the membrane fusion of autologous tumors with *Escherichia coli* cytoplasmic membrane using surgically‐derived mice TCMs, which contain all kinds of neoantigens produced during tumor occurrence and development, as the source of antigens.^[^ ^132^ ^]^ After three prophylactic vaccinations, specific CD8^+^ T cells in mice immunized with hybrid cell membrane‐coated NPs increased significantly, with all the mice infected with cancer cells presenting no tumors for at least 30 days.^[^ ^132^ ^]^ Although bacterial components are regarded as adjuvants for tumor vaccines, their toxicity and low yields will continue to inhibit their application in clinical treatment until such time when novel technologies capable of overcoming these hindrances will be developed.^[^ ^131^ ^]^

APC membranes serve as another alternative to hybridization with tumor cells for cancer vaccines. APCs’ surfaces contain various immune active factors, such as antigen peptide‐MHC complexes and co‐stimulatory molecules (e.g., B7 family members), which are the mainstay in anti‐tumor immunity initiation. Liu et al. extracted the DC‐tumor hybrid cell membrane (FM) after fusing DCs with treated tumor cells.^[^ ^133^ ^]^ Fortified with a nanoparticle core, NP@FM induced a salient boost in CTL formation through direct or indirect T cell activation.^[^ ^133^ ^]^ Additionally, the hybridized cell membrane‐coated NPs hybridized with nanophotosensitizers effectively killed primary and distant 4T1 tumors with PDT.^[^ ^134^ ^]^ Combined with ICBs, the DC‐tumor hybrid cell membrane considerably promoted tumor elimination and tumor re‐challenge rejection in immunotherapy.^[^ ^135^ ^]^ Unlike DCs, macrophages express more inflammatory factors and chemokine receptors, resulting in more robust immune responses and chemotaxis at inflammation sites. Macrophage‐tumor hybrid cell membranes show such properties as tumor antigen provision, inflammation site accumulation, and homogenous tumor targeting, enhancing the treatment of different tumors when combined with chemotherapy, microRNA (miRNA), and SDT.^[^ ^136^, ^137^, ^138^, ^139^ ^]^ However, APC‐generated endogenous antigens may provoke related autoimmunity or immune tolerance, and the molecules on APCs and tumors may interact with each other, influencing hybrid membrane function. The qualification and uniformization of the hybrid membrane, therefore, require further analysis.

Alongside APCs, erythrocytes have likewise been used as delivery vehicles to the spleen.^[^ ^140^ ^]^ Normal erythrocytes can eliminate bacteria in circulation and transfer their antigens to APCs in immune organs.^[^ ^141^ ^]^ Damaged erythrocytes, meanwhile, express phosphatidylserine (PS), which is an “eat me” signal mediating macrophage phagocytosis in the spleen; however, downregulating PS induces antigen presentation without the “eat me” signal.^[^ ^142^ ^]^ This suggests that combining or hybridizing tumor membrane vaccines with erythrocytes selectively activates splenic APCs and ignites general immunity against cancer. Han et al.’s erythrocyte‐tumor hybrid cell membrane, developed to successfully deliver tumor antigens to splenic APCs and activate adaptive immunity, thwarted the growth and metastasis of subcutaneous B16F10 and 4T1 tumors.^[^ ^143^ ^]^ Demonstrably, this hybrid system also adapts to brain tumor treatment. Shi, W. et al. coated fusion membranes extracted from erythrocytes and glioma cells U251 with isoliquiritigenin (ISL) to form ISL@HM NPs, which enhanced the solubility and bioavailability of ISL, bringing about more apoptotic U251 cell death.^[^ ^144^ ^]^

In summary, hybridizing tumor cell membranes with other cell membranes fuses the features of the components, maximizing their functions. Attention should now be turned to finding more membrane combinations with novel effects that can boost cancer treatment.

### 2.5 Addition of adjuvants

NPs with cancer cell membranes not only serve as antigen pools, but they also function as effective carriers of adjuvants. As common recipes of vaccines against viruses, adjuvants of Toll‐like receptor 3/7/8/9 (TLR3/7/8/9) agonists improve anti‐tumor immunity by effectuating type I interferon (IFN) response and CD8^+^T cell activation.^[^ ^92^ ^]^ Cytidine‐phospho‐guanosine oligonucleotides (CpG ODN), which are short single‐stranded synthetic DNA adjuvants, are broadly applied to camouflage pathogen‐associated molecular patterns (PAMPs), mobilizing immune cells through the triggering of the TLR9 signaling pathway in plasmacytoid DCs (pDCs) and B cells.^[^ ^145^ ^]^ Kroll et al. designed biodegradable poly(lactic‐co‐glycolic acid) (PLGA) NPs coated with B16‐F10 mouse melanoma‐derived membranes and CpG ODN 1826.^[^ ^146^ ^]^ Membrane wrappings altered the sizes and charges of the particles, facilitating target cell entrance. These NPs promoted antigen presentation and the co‐stimulatory molecule and cytokine production of bone marrow‐derived dendritic cells (BMDCs), strengthening tumor‐specific cellular immunity and achieving significantly higher survival rates in mice models treated with NPs combined with ICIs.^[^ ^146^ ^]^ In addition to melanoma‐derived membranes, the anti‐tumor effect of TCM vaccines is notably boosted by CpG ODN, as demonstrated by the more than 50% increase in the length of survival of mice in a C1498 acute myeloid leukemia (AML) model.^[^ ^147^ ^]^ As alternatives to CpG ODN, TLR7 agonists can replicate RNA virus stimulation and ignite robust cellular immune responses.^[^ ^148^ ^]^ Yang and coauthors’ poly(d,l‐lactide‐*co*‐glycolide) NP created by merging a mannose cancer cell membrane with the TLR7 agonist, imiquimod (R837), against B16‐F10 melanoma, upregulated the uptake of the NPs by DCs, CD103 expression on T cells, IFN‐γ levels in sera and the survival rate of B16‐F10 mice burdened with αPD‐1.^[^ ^50^ ^]^ Additionally, the TLR7/8 agonist, R848, has been proven to benefit tumor membrane vaccines, to hinder the progression of B16‐F10 and TC‐1 cancer by generating DC activation and M2‐like tumor‐associated macrophage (TAM) repolarization.^[^ ^149^ ^]^ In recent decades, TLR3/7/8/9 agonists have seen widespread investigation and deployment in cancer vaccines;^[^ ^150^, ^151^, ^152^, ^153^, ^154^ ^]^ however, ideal adjuvants or combinations of these agonists for cancer vaccines with TCM must still be uncovered.

As well as TLR agonists, metal agents are considered as crucial adjuvants in vaccines through their action on the innate immune pathway. Alexander Glenny's discovery of the promotion of the production of antibodies by alums nearly a century ago eventually led to the widespread use of aluminum salts. However, aluminum‐containing adjuvants’ enhancement of viral and cancer vaccines is limited by their unsatisfactory initiation of cellular immune responses.^[^ ^155^ ^]^ This fact highlights the importance of novel metal agent adjuvants for tumor vaccine applications. Manganese (Mn) is considered an excellent alternative owing to its biocompatibility and ability to activate cellular immunity by triggering the cGAS‐STING and NLRP3 pathways.^[^ ^156^, ^157^ ^]^ Furthermore, Mn significantly amplifies T1‐weighted magnetic resonance imaging (MRI) signals after binding to proteins, making it a suitable agent in cancer diagnosis.^[^ ^158^ ^]^ TCM coating of NPs with MnO_2_ apparently promotes cancer regression when deployed alongside radiotherapy, immunotherapy, photodynamic‐starvation therapy, chemotherapy drugs, and tumor suppressor genes.^[^ ^159^, ^160^, ^161^, ^162^, ^163^ ^]^ Combined with iron, silicon, and artemisinin, Mn strengthens MRI signals and the anti‐tumor immunity of cell membrane‐coated NP vaccines.^[^ ^164^, ^165^, ^166^ ^]^

## 3 TUMOR‐DERIVED EXTRACELLULAR VESICLES (TEVs)

**FIGURE 4:**
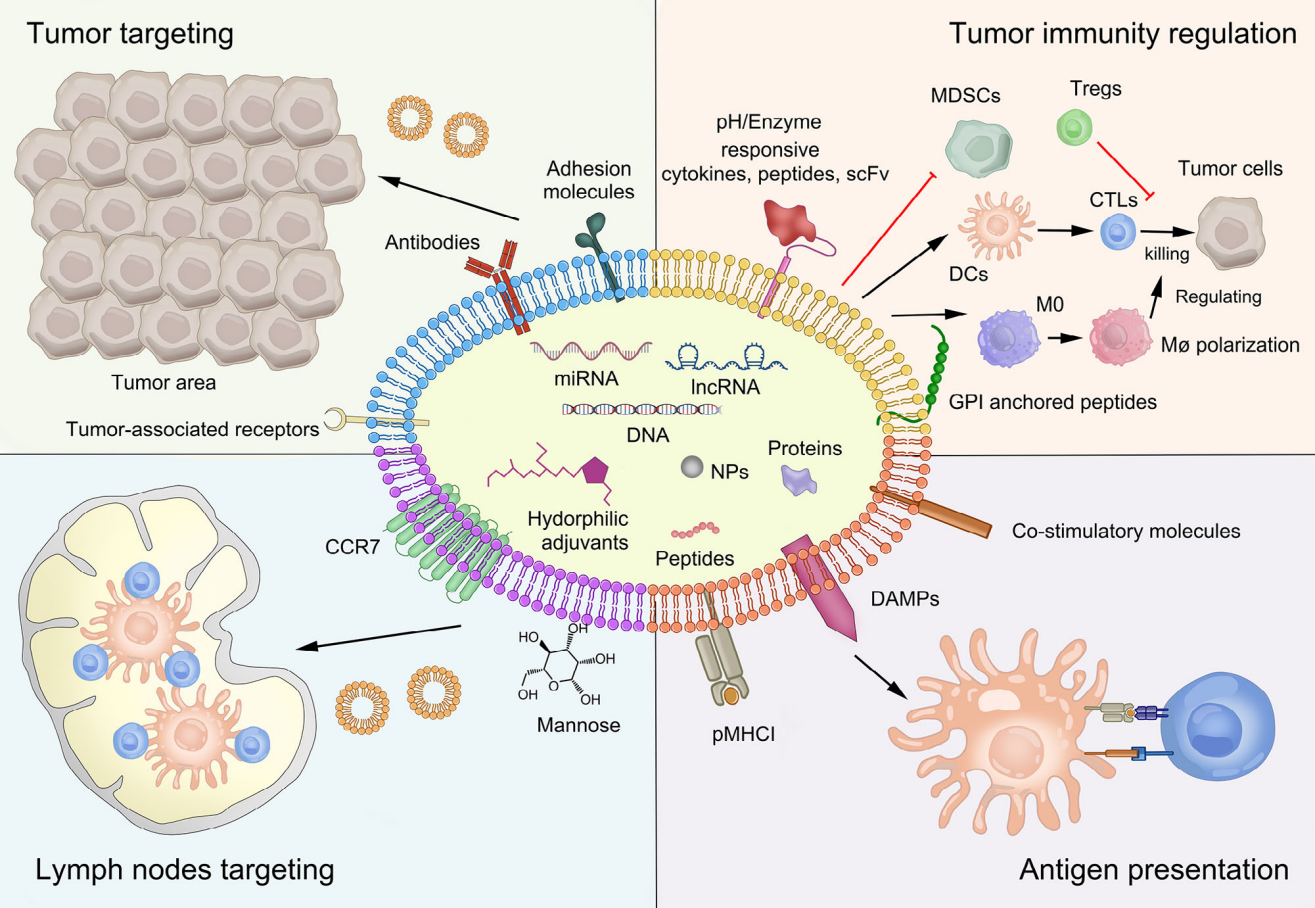
The inflammatory role of tumor‐derived extracellular vesicles (TEVs) in tumor microenvironment. The inherent factors of tumor can guide TEVs into tumor tissue or lymph nodes, which benefit targeting delivery and treatment. Owing to their high fabricability, various of anti‐tumor immunity enhancers can be modified on TEVs and accelerate tumor antigen presentation and reshaping of tumor immunological micro‐environment.

Extracellular vesicles (EVs), which are nano‐ or sub‐micron‐sized membrane‐derived vesicles generated from the blebbing of the cell envelope, are involved in many biological processes, such as intercellular communication and immunoregulation.^[^ ^167^ ^]^ Ranging from 50–2000  nm, EVs contain cytoplasmic components, like nucleic acids, proteins, and lipids, and can be categorized into eight subtypes according to size and origin: apoptotic bodies, ectosomes, filopodia‐derived vesicles, exophers, migrasomes, exosomes, extracellular matrix (ECM)‐bound vesicles, and arrestin‐domain‐containing protein 1 (ARRDC1)‐mediated microvesicles (ARMMs).^[^ ^168^ ^]^ Unlike synthetic nanocarriers, EVs are less toxic to healthy cells and carry the natural characteristics of cell membranes, for example, homotypic targeting, which helps increase the targeting properties of vaccines.^[^ ^169^ ^]^ Furthermore, the size of EVs expedites lymph node penetration and APC uptake.^[^ ^169^ ^]^ Tumor EVs (TEVs) are potential cancer vaccines owing to their richness in cancer information and DAMPs, activating DCs and tumor‐specific T cells to eliminate tumor cells. Thanks to the features of EVs, TEVs harbor many promising characteristics for cell‐free vaccine strategies, as well as an excellent plasticity property that can be engineered or modified towards functionalized vaccines with enhanced tumor targeting, lymph node targeting, antigen presentation, and anti‐tumor immunity (Figure 4). This section elucidates the detailed modification of tumor‐derived exosomes and microvesicles that are probed primarily in the application of EVs.

### 3.1 Tumor exosome (TEXs) vaccines

Derived from the fusion of multivesicular bodies (MVBs), exosomes are sorted as EVs with a diameter range of  30–150 nm and are secreted by multiple cell types.^[^ ^170^ ^]^ Generated in cells, exosomes contain a broad range of intracellular cargos, for instance, lipids, proteins, RNA, DNA, and metabolites, which participate extensively in many fundamental biological processes.^[^ ^171^ ^]^

Tumor cell‐generated exosomes (TEXs) are crucial to the occurrence, development, and metastasis of various cancers, for example, glioma, breast cancer, lung cancer, prostate cancer and melanoma, among others, as well as immune response and therapy resistance.^[^ ^172^, ^173^, ^174^, ^175^, ^176^, ^177^, ^178^, ^179^ ^]^ TEXs carry rich cancer information that includes MHC molecules, heat shock proteins (HSPs), tetraspanin (CD9, CD63, and CD81), immunosuppressive cytokines, and characteristic tumor antigens. Part of a key mechanism in the escape from the immune system, TGF‐β, Fas‐L, PD‐L1, NKG2D‐L, and TRAIL in tumors can be enveloped in TEXs and stymie the activation of T cells, NK cells, macrophages, and DCs with the promotion of regulatory T (Treg) cells and myeloid‐derived suppressor cell (MDSCs) infiltration.^[^ ^180^, ^181^, ^182^, ^183^, ^184^ ^]^ The MHC‐tumor antigen complex and HSPs in TEXs mobilize anti‐tumor immunity.^[^ ^185^, ^186^ ^]^ Additionally, exosomes from some tumor types can reside at their original tumor site, which naturally enhances the targetability of TEXs.^[^ ^187^ ^]^ Strategies to reconstruct TEXs are typically designed to intensify inflammatory factors and halt their immunosuppressive counterparts in TMEs based on this.

Genetic engineering can reshape TEXs to accelerate anti‐tumor immunity. As the first signals of immunological synapses, MHC molecules are core elements that initiate and maintain T cell activation. Expressed on APCs, MHCII is a key MHC member that mobilizes CD4^+^T cells. Several studies have focused on scrutinizing TEXs from tumor cells transduced with the MHC II transactivator protein CIITA (Class II transactivator) gene.^[^ ^188^, ^189^ ^]^ These TEXs vaccines, made from DCs, were observed to upregulate MHC II and CD80 expression, heightening the production of inflammatory cytokines such as TNF‐α, IFN‐γ, and IL‐12. Besides, co‐stimulatory molecules are excellent inserted factors as secondary signals of immunological synapses. Li et al. found that exosomes from the murine leukemia cell L1210 failed to boost CD4^+^T cells on account of L1210's low co‐stimulatory molecule capacity. Therefore, the researchers genetically modified L1210 expressing CD80 and CD86, and the exosomes from the new L1210 facilitated CD4^+^T cell proliferation and Th1 cytokine production, resulting in significant tumor inhibition and prolonged survival time in the examined mouse model.^[^ ^190^ ^]^ Cytokines and immune checkpoints are also key factors boosting T cell regulation. MC38 tumor‐derived exosomes with IL‐12 overexpression and TGF‐β1 knockdown enhanced DC‐based therapy and inhibited tumor growth via the activation of Th1.^[^ ^191^ ^]^ To abrogate the impact of immune checkpoints on anti‐tumor immunity, Huang et al. developed exosomes from acute lymphocytic leukemia cells with silenced PD‐L1 (LEX_PDL1si_).^[^ ^192^ ^]^ In their analyses, they found that LEX_PDL1si_ ignited a vigorous immune response with enhanced DC maturation, T cell proliferation, and Th1 cytokine release, and half the number of mice with LEX_PDL1si_ immunization survived for 30 days after they were infected with L1210 leukemia cells.^[^ ^192^ ^]^

The surface of TEXs comprises largely of lipid bilayers, which enable the delivery of desired cargoes, like adjuvants, drugs, and NPs. Some chemotherapeutic drugs, like doxorubicin (DOX), paclitaxel, cisplatin, and methotrexate, were designed to be wrapped in TEXs, and these complexes have been shown to engineer better therapeutic efficiency and cause diminished side effects.^[^ ^193^ ^]^ Also, pathogenic organismic derivations can serve as adjuvants for TEXs, boosting anti‐tumor immunity. Staphylococcal enterotoxin A (SEA) is demonstrably a satisfactory murine lymphoma‐derived exosome additive with increased IL‐2 and IFN‐γ production of T cells and an accelerated CTL response.^[^ ^194^ ^]^ The G protein of the vesicular stomatitis virus (VSV‐G) is an excellent accelerator of the internalization of TEXs by target cells, for it can mediate the attachment of viruses to cell surfaces. As a viral element, VSV‐G on TEXs is recognized as PAMPs promoting DC maturation by upregulating CD80, CD86, CD40, and IL‐12.^[^ ^195^ ^]^ The H162R mutation of VSV‐G has noticeably accelerated membrane fusion at the pH of TMEs.^[^ ^196^ ^]^ Kim et al. successfully ignited and propagated robust anti‐tumor immunity using TEXs made with a H162R mutant expressing VSV‐G (extracted from VSV‐G overexpressing tumor cells made by genetic engineering) on the surface.^[^ ^197^ ^]^ To prevent tumor cell immune escape, Wu L. et al. loaded B16F10‐derived exosomes with sodium polytungstate and metformin, inhibiting CD39 and activating AMP‐activated protein kinases.^[^ ^198^ ^]^ This drug complex impeded the progression and distant metastases of B16F10, while prompting more memory T cells to prevent tumor recurrence. In addition to carrying small molecules, TEXs can be paired with NPs, such as gold NPs. TEX‐wrapped gold NPs (Au@MC38) produced using tumor cells cultured in a medium containing HAuCl_4_ solution were found to cause aggravated ICD, resulting in the boosting of immunity.^[^ ^199^ ^]^

Despite some advances in TEX modification approaches, inserting peptides and proteins stably and effectively via gene engineering and chemical reactions remains a challenge. The indeterminacy of transfection efficiency limits the application of genetic engineering in parental tumor cells, whereas chemical modifications are restricted by the features of additional molecules, and some chemical functionalization, for instance, click chemistry, may decompose the surface structure. Finding guide molecules that specifically combine the characteristics of exosome proteins is critical. Through phage display techniques, Gao et al. developed a peptide, CP05 that specifically binds to CD63 on exosomes, and conjugating a muscle targeting peptide to CP05 showed increasing dystrophin expression and muscle function in an animal model.^[^ ^200^ ^]^ The discovery of exosome‐guiding peptides boosts drug‐loading exosome therapies in bone regeneration,^[^ ^201^, ^202^, ^203^ ^]^ myocardial infarction‐ischemia/reperfusion,^[^ ^204^ ^]^ exosome source identification,^[^ ^205^ ^]^ and chemo/photodynamic therapy enhancement.^[^ ^206^ ^]^ Based on this, CP05 is an excellent linker between TEXs and additional molecules that can amplify anti‐tumor effects.

As an alternative to TEX treatment, DCs loaded with TEXs markedly foster anti‐tumor immunity responses, given that DCs provide natural costimulatory molecules, inflammatory cytokines, and abundant MHC molecules, which are usually insufficient for T cell activation on tumors. The activation of CTLs with increased IFN‐γ production by melanoma TEX‐loaded DCs was first reported by Wolfers et al.^[^ ^207^ ^]^ Unlike tumor lysates, TEXs contain complete membrane constructions and richer contents, which activate DCs and induce significant CTL cytotoxicity without leading to DC apoptosis.^[^ ^208^, ^209^, ^210^, ^211^ ^]^ Additionally, TEXs serve as effective agents for DC imaging, and tracking TEXs is easy for DC internalization.^[^ ^212^ ^]^

### 3.2 Tumor derived‐microvesicle (TMV) vaccines

Microparticles (MPs), with a diameter range of 100–1000 nm, are heterogeneous plasma membrane microstructures that are released into the extracellular space by cells in response to stimuli such as hypoxia, injury, and pressure.^[^ ^213^ ^]^ MPs reportedly express some characteristic markers, for example, phosphatidylserine, CD40, matrix metalloproteinases (MMPs), selectins, integrins, and ADP‐ribosylation factor 6 (ARF6), thanks to their plasma membrane origin.^[^ ^214^ ^]^ Distinct from exosomes, microparticles are mainly generated from the budding and fission of the plasma membrane and contain different components^[^ ^167^, ^215^ ^]^ (Table 2). Microparticles have a larger volume to carry more engineered products and can be separated with lower centrifugal speed compared with exosomes, which reduces the difficulty of manufacture and clinical transformation.

**TABLE 2:** Differences between exosomes and microparticles.

	Exosomes	Microparticles
Origin	Endosomal pathways	Budding and fission of plasma membrane
Size	30–150 nm	100–1000 nm
Contents of membrane	CD9, CD81, CD63, CD151, CD37, CD53, TSPAN6, TSPAN8	CD9, CD81, CD82
Lipids	Phosphatidylserine cholesterol, ceramide, sphingolipid	Phosphatidylserine, phosphatidylethanolamine, sphingolipid
Cell adhesion	Intergrin, lactadherin, ICAM	Intergrin, PECAM1, fibronectin
Signal transduction	Protein kinases, β‐catenin, 14‐3‐3	Protein kinases, β‐catenin, 14‐3‐3, ARF6,RAB11, ROCK
Cytoplasmic material	GAPDH	Tau, TDP43, GAPDH
Cytoskeleton molecules	Lack of cytoskeleton molecules	Actin, tubulin
Biogenesis factors	ALIX, TSG101, syntenin, ubiquitin, clathrin, VPS32, VPS4	ALIX, TSG101, ERK, PLD, VPS4
Nucleic acids	mRNA, DNA, microRNA, non‐coding RNA	mRNA, DNA, microRNA, non‐coding RNA

Abbreviations: ALIX, ALG‐2 interacting protein X; ARF6, ADP‐ribosylation factor 6; ERK, extracellular regulated protein kinases; GAPDH, glyceraldehyde‐3‐phosphate dehydrogenase; ICAM, intercellular cell adhesion molecule; PECAM1, platelet endothelial cell adhesion molecule 1; PLD, phospholipase D; ROCK, RHO‐associated protein kinase; TDP43, TAR DNA‐binding protein 43; TSG101, tumour susceptibility gene 101 protein; TSPAN, tetraspanin; VPS, vacuolar protein sorting‐associated protein.

TMVs are common particles in TMEs and probably contribute to cancer progression. The characteristics and functions of TMVs are gradually and vastly being unveiled with progressively more focused investigations. As facilitators of tumor enhancement, TMVs can directly promote angiogenesis, invasion, and tumor metastasis, as well as interfere with anti‐tumor immunity in TMEs.^[^ ^216^, ^217^ ^]^ TAMs can be polarized into M2 macrophages and proliferate after TMV uptake.^[^ ^218^ ^]^ However, reshaping immunity in TMEs is a double‐edge sword for TMVs. TAMs and DCs can be stimulated by TMVs via the cGAS‐STING‐TBK1‐STAT6 pathway, leading to inflammatory factor production,^[^ ^218^, ^219^, ^220^ ^]^ and this makes the application of TMV vaccines possible. Moreover, changes in the pH of lysosomes result in the regulation of macrophage polarization, implying that delivering drugs that modify lysosome acidity possibly mitigates M2 macrophage induction.^[^ ^221^ ^]^

Elevating the production of TMVs is vital for TMVs vaccines because tumor cells secrete little MVs in a normal state. Previous studies have demonstrated the TMV‐boosting way by external stimuli such as mechanical extrusion, acoustic stimulation, electric stimulation, glycolysis and oxidative phosphorylation inhibition, hypoxia, acidity, starvation, and liposome addition.^[^ ^222^ ^]^ To produce TMV vaccines for tumor, the TMV boosting ways of inducing tumor cell stress and damage can promote the generation of DAMPs that activate immune pathways. Chemotherapy with particular agents can initiate and accelerate ICD in tumor cells, mainly by protein kinase R (PKR)‐like endoplasmic reticulum kinase (PERK) signaling and ROS production. Anthracyclines have been proven to enhance p53 expression, which activates PERK signaling and EIF2α phosphorylation, leading to ER stress (also termed integrated stress response, ISR), whereas metal‐based chemicals induce ROS, igniting oxidative stress.^[^ ^223^ ^]^ All of these products can ignite inflammatory pathways and promote neoantigen generation when they are captured by APCs.^[^ ^224^ ^]^ However, many studies focus on tumor membrane carrying chemotherapeutics for cancer treatment, yet membranes of chemically pre‐incubated tumor cells have not received considerable attention. Besides chemotherapy, some elements of ROS can also strongly trigger ICD, namely type 2 inducers.^[^ ^223^ ^]^ Hypochlorous acid (HOCl) is one of the most essential participants in ROS and protects bacteria from invasion during acute inflammation.^[^ ^225^ ^]^ Early studies showed that DCs activated by HOCl‐treated antigens and tumor cells set up a more violent T cell response, resulting in improved therapeutic outcomes in mouse model and patients.^[^ ^226^, ^227^, ^228^, ^229^ ^]^ Mechanically, HOCl and sequencing ROS reactions augment HLA presentation in monocyte‐derived DCs (mo‐DCs), leading to stronger T cell priming.^[^ ^230^ ^]^ According to the properties of immunogenicity promotion, HOCl can serve as a good preconditioner of tumor cell membranes and derivations. Y. H. Zhou et al. designed a melittin‐encapsulated hydrogel scaffold loading secretion of HOCl‐conditioned B16F10 cells (HOCl‐CDS hydrogel) to help B16F10 cell secretion release in tumor site.^[^ ^231^ ^]^ These secretions, including TMVs, highly activated the type Ι IFN pathway and enhanced the expression of B7‐1/2. In an in vivo experiment, HOCl‐CDS hydrogel protected more than half of the mice from death with growing number of IFN‐γ^+^ T cells and declined M1/M2 ratio.

**FIGURE 5:**
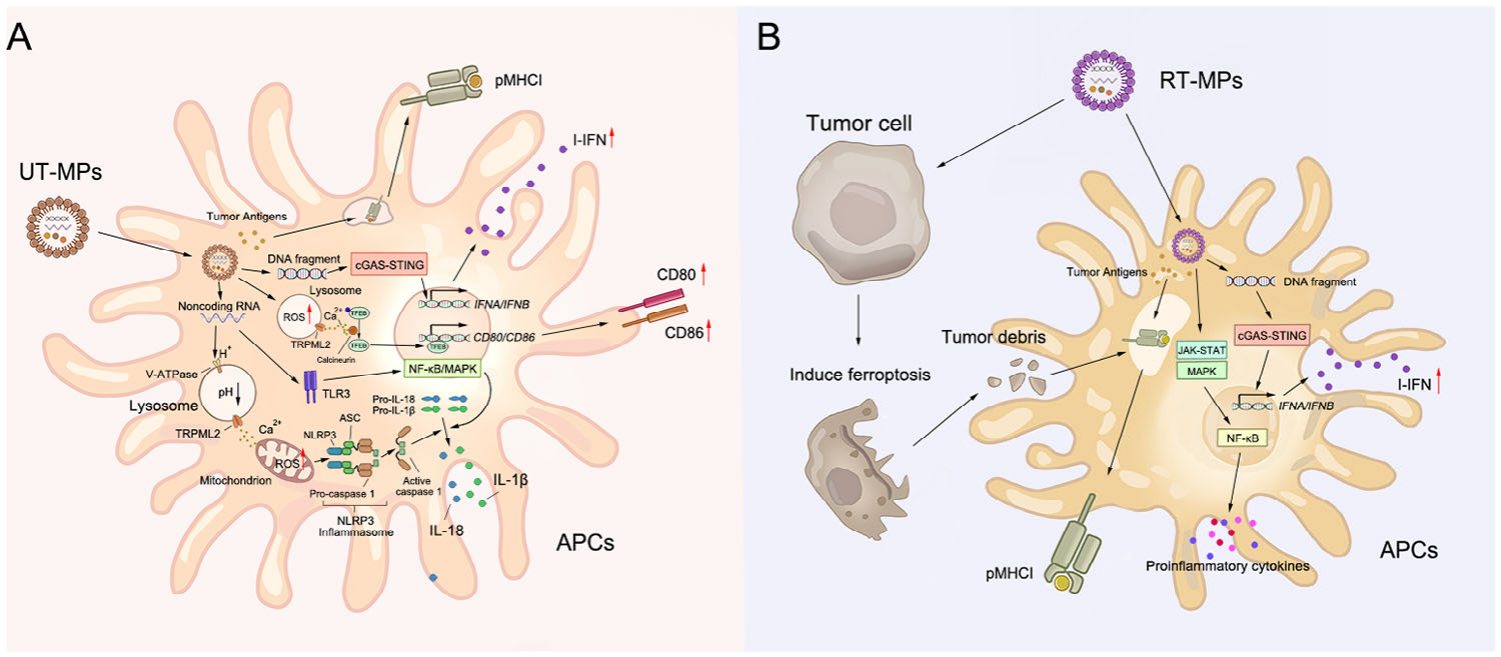
The effects of (A) UV‐treated tumor cell MPs (UT‐MPs) and (B) irradiated tumor cell‐released microparticles (RT‐MPs) on APCs.

Ultraviolet (UV) light and radiation can also boost the production of TMVs with antigens, DNA fragments, noncoding RNA and other DAMPs that have effects on APCs by distinct mechanisms (Figure 5). For several decades, UV has been regarded as a common method to produce cancer vaccine because it can prevent tumor proliferation and facilitate ICD via DNA damage and ROS generation.^[^ ^232^ ^]^ However, UV's therapeutic effects are not as satisfactory as expected. Besides causing cell death, UV is a proven outstanding strategy to promote the release of MPs from tumors. Isolated from tumor supernatants, UV‐treated tumor cell MPs (UT‐MPs) contain various tumor antigens, DNA, RNA, and proteins which can regulate inflammatory pathway of APCs (Figure 5A). When loaded with CpG and Fe_3_O_4_, UT‐MPs can polarize TAMs into M1 macrophages and inhibit ∼83% of B16F10 tumor progression in mice.^[^ ^233^ ^]^ Captured by macrophages, the endocytosis of UT‐MPs reduces lysosomal pH and upregulates ROS via Ca^2+^ transfer, activating NLRP3 inflammasome.^[^ ^221^, ^234^ ^]^ UT‐MPs can also be taken up by DCs, and DNA fragments in MPs strongly accelerate IFN‐I secretion via the cGAS‐STING pathway.^[^ ^219^ ^]^ Additionally, UT‐MPs have been shown to boost the expression of NADPH oxidase 2 (NOX2) in DCs, mediating Ca^2+^ outflow from lysosomes, which dephosphorylates the transcription factor EB (TFEB), prompting its binding to the promoters of CD80 and CD86 and resulting in the increased expression of CD80 and CD86.^[^ ^235^ ^]^ Multiple studies have shown that UT‐MPs inhibit tumor growth by promoting DC chemotaxis, boosting DC, and enhancing CD8^+^T cell infiltration in TMEs.^[^ ^217^ ^]^ As carriers of biogenesis, UT‐MPs improve the therapeutic effect of chemotherapy agents, such as DOX, cisplatin, and methotrexate (MTX).^[^ ^236^, ^237^, ^238^ ^]^ However, UT‐MP treatment has some shortcomings: non‐coding RNAs in MPs can polarize macrophages into M2 phenotypes by activating the TLR3‐MAPK‐NFκB pathway.^[^ ^239^ ^]^ Further research must be performed to uncover the mechanism of regulation by UT‐MPs in different cell types. As an alternative method to UV, radiation can be deployed in TMV induction via oxidative stress and DNA damage, igniting stronger cGAS‐STING signaling activation and the radiation‐induced bystander effect (RIBE).^[^ ^240^, ^241^ ^]^ Cao et al. found that irradiated tumor cell‐released microparticles (RT‐MPs) enhanced anti‐tumor immunity and triggered ICD in a malignant pleural effusion (MPE) model largely through ferroptosis.^[^ ^242^ ^]^ The examined exogenously‐injected TMVs were engulfed by TAMs and reshaped into M1 phenotypes via JAK‐STAT and MAPK pathways in TME, fortifying tumor immunosurveillance^[^ ^242^ ^]^ (Figure 5B). In a glioblastoma model, irradiated C6 TMVs displayed similar effects, including increased cytotoxic T cell infiltration and more than 50% tumor volume reduction, on tumor growth.^[^ ^243^ ^]^ The spike protein and TGFBR2 overexpressing‐RT‐MPs designed using genetic engineering inhibited the progression of Lewis LLC by activating DCs and macrophages and neutralizing TGF‐β.^[^ ^244^ ^]^ Besides UV and radiation, microwave ablation (MWA) is also effective to induce ICD of tumor cell via high temperature leading protein denaturation, enzyme inactivation, DNA damage, and mitochondrial disorder, which produce DAMPs. Previous studies showed that MWA can trigger ferroptosis by induction of related factors such as ROS, HSPs, hypoxia‐inducible factor (HIF), p53, and nuclear factor erythroid 2‐related factor 2 (NRF2).^[^ ^245^ ^]^ However, there is still no evidence of the anti‐tumor ability of TMVs inducing MWA.

Because of their size and still developing extraction process, TMV injections face safety problems. As an alternative means of administration, the oral route has the advantages of safety, acceptance by patients, and mucosal immune response induction. TMV oral vaccines are designed from B16F10 and CT26 tumor cells mobilizing CD103^+^CD11c^+^ DCs by intestinal epithelial cells whose NOD2, MAPK, and NF‐κB pathways were activated and inflammatory chemokines were expressed.^[^ ^246^ ^]^ Additionally, oral microvesicle vaccines developed from murine prostate cancer cells yielded a 5‐fold reduction in tumor volume when combined with GM‐CSF.^[^ ^247^ ^]^

Based on recent findings, TEVs are ideal carriers of tumor antigens with the addition of anti‐tumor factors, such as cytokines, adjuvants, and nucleic acids, representing an exceptional tumor membrane‐derived vaccine for cancer treatment. However, various TEV components require further evaluation, and mounting obstacles in areas, like the characterization, purification, and magnified production of large‐scale applications, must still be overcome.^[^ ^248^ ^]^ Also, distinguishing TMVs from TEXs based on their sizes, whose ranges overlap, is hard, and there is still no common biomarker for identifying TEVs for isolation. Furthermore, the standards of TEV production and classification for biopharmaceuticals have yet to be established, and this prevents the clinical application of TEV vaccines. To overcome these hurdles, research to improve TEV engineering and production in tandem with the establishment of quality control is underway, with the findings potentially setting the basis for clinical practice.

## 4 TCM VACCINES AND IMMUNE CHECKPOINT THERAPY (ICT)

Immune checkpoint therapy (ICT), by blocking immunosuppressive T cell signals in TMEs, has revolutionized cancer immunotherapy with relatively higher efficiency in tumor elimination. Combined with traditional treatments like chemotherapy, radiotherapy, surgery, and other immunotherapies, ICT has successfully prolonged the survival time of numerous patients with various tumor types and has even cured some patients.^[^ ^249^ ^]^ Nevertheless, resistance to ICT is universal in certain types of cancers, including pancreatic carcinoma and glioblastoma, while adaptive resistance may occur in a number of patients with ICT‐sensitive tumors during long‐term treatment.^[^ ^250^ ^]^ To elucidate why the effectiveness of ICIs varies from patient to patient, researchers built a cancer immunogram to sum up vital factors for immunotherapy: tumor foreignness, general immune status, immune cell infiltration, absence of checkpoints, absence of soluble inhibitors, absence of inhibitory tumor metabolism, and tumor sensitivity to immune effectors.^[^ ^251^ ^]^

Complementing ICT, vaccines developed from tumor membranes and their derivatives can provide numerous antigens with ample tumor information to APCs, resulting in the elevated expression of tumor‐related peptide‐loaded MHC I complexes, enhancing tumor immunogenicity, and promoting tumor‐specific CTL infiltration. Furthermore, tumor membrane modifications speed up TME remodeling by amplifying inflammatory factors and diminishing tumor‐promoting molecules. For instance, anti‐PD‐1 mAb failed to save mice burdened with MOC1 120 days after tumor inoculation, whereas two‐fifths of the mice survived when treated with a tumor membrane vaccine carrying GPI‐anchored IL‐12 and CD80.^[^ ^125^ ^]^ A tumor membrane vaccine built from CD47KO/calreticulin dual‐bioengineered B16F10 cells was shown to activate the “eat me” signal of macrophages and induce ICD.^[^ ^252^ ^]^ Alongside α‐PD‐L1, the vaccine suppressed tumor progression and facilitated CTL infiltration better than single α‐PD‐L1 therapy.^[^ ^252^ ^]^ In addition to participating in engineering, tumor membranes can act as carriers for chemotherapy, magnetic hyperthermia, photothermal therapy, and photodynamic therapy in synergy with ICT.^[^ ^51^, ^253^, ^254^, ^255^, ^256^ ^]^ As well as direct tumor membrane delivery, tumor cells can undergo significant inhibition by NPs wrapped in the membranes of DCs, which pre‐pulses tumor membrane NPs.^[^ ^257^ ^]^ This cascade cell membrane system avoids the negative effects of immunosuppressive factors on tumor membranes and demonstrably strengthens the potency of ICIs in transplanted Hepa1‐6, B16F10, and TC‐1 tumor models.^[^ ^257^ ^]^

While tumor membrane vaccines benefit ICT, ICI treatment has a long way to go with the challenges they face. Long‐term ICT use could cause immune‐related adverse events (irAEs) consisting of more than 70 diverse pathologies that implicate almost every organ.^[^ ^250^ ^]^ Evidence suggests that tissue‐resident memory CD8^+^T cells, neutrophils, IL‐6, and IFN‐γ play vital roles in ICT‐related colitis, while memory CD4^+^T cells are key to initiating destructive thyroiditis and encephalitis.^[^ ^258^, ^259^, ^260^, ^261^ ^]^ The appearance of irAEs may not be universally identical, and the mechanisms have not been fully understood yet. Further inquiries are required to set the foundation for improved delivery systems by TCMs to alleviate irAEs.

## 5 DISCUSSIONS

This review provides an overview of the progress in and application of TCM vaccines and their derivatives in cancer treatment. Therapeutic anti‐tumor vaccines serve to mobilize the immune system to hinder malignant tumor proliferation and expand the repertoire of antigens recognized by memory cells. To achieve this goal, certain tumor‐specific antigens are indispensable to the stimulation of DCs for immune recognition and T‐cell activation. TCM complexes function as natural antigen pools from which abundant antigens ensuring the effective recognition of heterogeneous tumor microenvironments can be derived. Regarding vaccine platforms, TCMs also serve as ideal carriers owing to their abundant protein receptors and ligands on the surface and ease of functional modifications. Benefiting from proper surface functionalities, including biological engineering, physical modification, or chemical connection, and associated with some natural or artificial adjuvants, membrane‐based vaccines are capable of lymph node targeting, tumor homing, immune and microenvironment modulation, as well as activating specific immune killing effects and enhancing the effects of ICT in tumor eradiation. Nevertheless, multiple challenges, including but not limited to elevating cytotoxicity against tumor cells, reducing adverse events, and process standardization, must still be overcome to improve tumor treatment efficiency.

Cold tumors, with paucity or exclusion of T cells in TMEs, are among the most common causes of resistance to tumor immunotherapy.^[^ ^262^ ^]^ Mechanically, the absence of MHC class I is critical to facilitating cold tumor escape from immunosurveillance through a defect in tumor antigen exhibition and presentation.^[^ ^263^ ^]^ Insufficient MHC class I and tumor peptide complexes hamper the efficiency of cancer vaccines, which sometimes even downgrades the abundance of MHC class I further.^[^ ^264^ ^]^ These facts show the importance of strategies that boost tumor cell MHC class I. To raise tumor cell MHC class I expression in patients and cell membrane donors, the mechanism through which tumor cells are inhibited by MHC I‐peptide complex formations requires comprehensive elucidation. Endogenous antigen processing and surface presentation (APSP) are orchestrated by APSP‐related proteins, such as β2‐microglobulin (B_2_M), transporters associated with antigen processing (TAPs), proteasome subunit beta types (PSMBs), and ER amino peptidases (ERAPs).^[^ ^265^ ^]^ In Lewis lung carcinoma LLC1 and glioma GL261, the genes expression of APSP‐related proteins was proven to be changed, giving rise to the dysfunction of T cells to recognize tumor cells under DC vaccine therapy.^[^ ^266^ ^]^ In another research, TNF receptor‐associated factors 3 (TRAF3) acted as a negative regulator of MHC class I by suppressing the NF‐κB pathway, and TRAF3 inhibition upregulated MHC class I, resulting in T cell cytotoxicity sensitization and ICT promotion.^[^ ^267^ ^]^ The spectrum of tumor APSP regulators and related antigens remains incomplete, and novel detection technologies of biochemistry,^[^ ^268^ ^]^ next generation sequencing and silicon prediction^[^ ^265^ ^]^ could help put together all the pieces of the puzzle, which would instruct the engineering of TCM vaccines with stronger immunogenicity. Besides regulating APSP‐related proteins, some chemicals have been found to function as MHC class I enhancers. Fan et al.’s dual PI3K/HDAC inhibitor, BEBT‐908, suppresses multiple tumor types through the upregulation of MHC class I in a STAT1‐dependent manner.^[^ ^269^ ^]^ This result implies that MHC class I enhancer agents can be used directly in membrane donor tumors or be wrapped in TCMs to act on tumors in patients, and these are potent strategies to boost the efficiency of cancer vaccines.

Despite its abundance in the MHC I‐peptide complex, it is only one of the multiple influencing factors of tumor immunogenicity that determines the intensity of anti‐tumor immunity. ICD plays a key role in mobilizing anti‐tumor immune responses via the release of DAMPs, including host nucleic acid, ATP, high mobility group protein 1 (HMGB1), and PAMPs, which can be recognized by receptors of immune cells.^[^ ^270^ ^]^ Some regulated cell death (RCD) types, like autophagy, ferroptosis, pyroptosis, and necroptosis, trigger ICD to trigger anti‐tumor immunity through participation in the survival, differentiation, activation, transport, and functioning of immunocytes.^[^ ^271^ ^]^ Several agents targeting or engendering these RCDs have been applied clinically, providing a basis for the modification of tumor membrane vaccine donor cells. Nevertheless, RCDs also allegedly antagonize anti‐tumor immunity under certain circumstances; for instance, MDSCs, Treg cells, and M2 macrophages can be activated by the RCD.^[^ ^271^ ^]^ To amplify immune synergism and diminish tumor promotion, UV irradiation, radiotherapy, nanotechnology, bioinformatics, and silicon should be considered for new strategies in the induction of ICD via the RCD,^[^ ^232^, ^272^, ^273^, ^274^ ^]^ which should benefit the modification of tumor membrane vaccines.

The prerequisite for high efficiency of tumor membrane vaccines is not only strong donor immunogenicity, but also efficient and specific absorption in target sites. DCs and macrophages in immune organs are considered as ideal targets because they engage in the uptake, processing, and T‐cell priming of tumor antigens. As demonstrated in previous studies, senescent or damaged erythrocytes target splenic macrophages and DCs.^[^ ^275^ ^]^ Furthermore, high CD47 expression on erythrocytes frustrates the clearance of reticuloendothelial cells and macrophages, leading to prolonged circulation.^[^ ^128^, ^276^ ^]^ The characteristic of splenic APC targeting facilitates the application of hybridized cell membrane vaccines developed from tumor membranes and erythrocytes. In addition to the spleen, draining lymph nodes (LNs) function as outposts for immunocytes associated with tumors. NPs with the chemokine C‐C motif ligand 21 (CCL21), ferritin, and melittin, along with lipids, have been reported as APC targeting in LNs, accelerating tumor remission.^[^ ^277^, ^278^, ^279^ ^]^ As carriers of mRNA vaccines, lipid nanoparticles (LNPs) made from 113‐O12B have shown outstanding LN‐targeting specificity and remarkably elevated CD8^+^ T cell cytotoxicity with OVA‐encoding RNA in a B16F10‐OVA tumor model.^[^ ^280^ ^]^ All the results above can offer acceptable strategies capable of enhancing tumor membrane vaccines. However, large particles (>30nm) via LN‐targeting delivery can be shielded and captured by the LN reticular network, especially macrophages in the subcapsular sinus (SCS) floor.^[^ ^281^ ^]^ SCS macrophage depletion smooths the effectiveness of vaccines;^[^ ^282^ ^]^ yet destroying the LN barrier could likely influence the micro‐environment of LNs and result in the loss of protection from harmful immunogenic molecules.^[^ ^283^ ^]^ To avoid the phagocytosis of SCS macrophages, Schudel et al. designed a synthetic nanoparticle carrier system that enters lymph vessels and releases its cargo before macrophage uptake.^[^ ^284^ ^]^ This system escapes the hinderance of SCS via decomposing into small molecules instead of SCS suppression, which is realized by retro‐Diels‐Alder reaction of thiol‐reactive oxanorbornadiene (OND) linkers.^[^ ^284^ ^]^ The platform could mediate the spread of tumor membrane vaccines into the cortex and paracortex of LNs. In addition to secondary lymphoid organs (SLOs), TLSs serve as the most straightforward centers of immune cells in tumors and are associated with tumor progression and response to immunotherapy.^[^ ^285^ ^]^ The formation of TLSs can be divided into three steps: initial local fibroblast activation by tumor specific T and B cells, recruitment of immunocytes, and maturation.^[^ ^286^ ^]^ Anatomically, unlike SLOs, SLEs lack encapsulation, facilitating the delivery of macromolecules without phagocytosis.^[^ ^285^ ^]^ Further analyses should unveil the unknown mechanism of SLE formation and help find ideal agents capable of inducing SLEs in tumors, which would boost tumor membrane vaccine therapy.

TCM vaccines are competitive with current vaccines in clinics, such as mRNA vaccines. Since great success has been achieved in protecting COVID‐19, many studies are involved in the development of mRNA vaccines, which can be made into two forms: non‐replicating mRNA and self‐amplifying mRNA (saRNA).^[^ ^287^, ^288^ ^]^ Conventional mRNA (non‐replicating mRNA) is prepared by in vitro transcription (IVT) or chemical synthesis, loaded via NPs or LNPs.^[^ ^289^ ^]^ saRNA comprises of a part of alphavirus genome containing nsP1‐4 and RNA sequence coding target molecules. The complex of nsP1‐4 can be assembled into RNA‐dependent RNA polymerase (RdRP) which mediates the production of subgenomic RNA from RNA sequence of interest.^[^ ^290^ ^]^ All two kinds of RNA vaccines show higher antigen expression compared with vaccines made of proteins or peptides including TCM. However, external mRNA or alphavirus‐derived RdRP themselves have strong immunogenicity that are easier excluded by immune cells or pathways although some efforts have been put into reducing the immunogenicity.^[^ ^291^, ^292^, ^293^ ^]^ Besides, the discovery of SARS‐CoV‐2 fragment insertion into chromosome DNA implied the risk of gene integration by reverse transcriptional products of mRNA vaccines.^[^ ^294^ ^]^ Recently, Mulroney et al. found that +1 ribosome frameshifting is caused by N1‐methylpseudouridylation which is a protective modification of mRNA, indicating some safety problems of mRNA vaccine application.^[^ ^295^ ^]^ In comparison, TCM vaccines mainly contain proteins of tumor without potential toxicity of RNA. With the ability to act as an agent carrier, TCM and its derivation can load mRNA vaccines for the synergism of cancer protection and treatment. Wenqi S. et al. developed a mRNA vaccine and the sonosensitizer Ce6 loaded by 4T1 TCM coating PLGA‐NPs, which significantly inhibit the progression of tumors combined with ultrasound radiation.^[^ ^296^ ^]^ This study demonstrated the feasibility of the combination of the mRNA vaccine and TCM. In comparison, TEVs are a more appropriate carrier of mRNA. Nevertheless, there is still no study or application of TEVs loading mRNA vaccines because ribonuclease in TEVs can mediate RNA degradation. Some obstacles impede the development of NPs based TCM and mRNA‐combined vaccines. The properties of different NPs are not fully understood, leading to unknown adverse effects such as chronic inflammation and non‐target toxicity. Besides, the aggregation of some NPs may cause vascular embolism and thrombosis.^[^ ^289^ ^]^

Based on the arrangement and reorganization of TCMs, TMVs encapsulate parts of cytoplasmic functional biomolecules and act as multifunctional carriers without relying on the coating on other nanomaterials after combining pre‐secreting and post secreting functional strategies, including surface modification, gene engineering, component encapsulation, or membrane fusion. Nevertheless, the industrial manufacture and clinical transformation of TMVs face challenges, such as the imperfect GMP standards, avoiding risks of ancillary contamination, quantity, quality control, and animal preclinical models.^[^ ^297^ ^]^ In order to more accurately define MVs and their functions and to properly merge the methods for isolating MVs, the International Society for Extracellular Vesicles (ISEV) has issued a detailed guideline.^[^ ^298^ ^]^ These criteria cover every process, from cell culture to vesicle isolation and identification, thus ensuring the quality of macrovesicles extracted by different laboratories to some extent. At present, the principal methods for extracting extracellular vesicles include ultracentrifugation, density gradient separation, sonication, and extrusion via porous membranes. All these approaches are time‐consuming and cumbersome and often require a large number of cells or excessive amounts of culture supernatant. They are, therefore, possibly more suitable for preclinical rather than clinical research. Additionally, the main donors of antigens obtained from autogenic sources are resected tumor tissues or biopsy samples, which can only provide limited tumor cells for *ex vivo* proliferation. One way to increase MV yields is to use microfluidics technology, which enables the sophisticated control of submicrometer particles in small sample volumes and has been proven to separate tumor cells, bacteria, and microspheres effectively.^[^ ^299^ ^]^ Another feasible solution to improve macrovesicle yields is to simulate the process of virus exocytosis and promote the effective efflux of MVs by expressing VSVG‐like proteins on the surface of MVs.^[^ ^300^ ^]^ Spurred on by the delivery mechanism of vesicular stomatitis and the proficient membrane fusing activity of VSV‐G combined with the split green fluorescent protein (GFP) system, Zhang et al. built a novel vesicle system that specifically expresses any protein of interest on membranes with 1000‐fold higher yields than the negative control group.^[^ ^301^ ^]^

TCM is a fundamental cell component with remarkable biocompatibility. As TCM vaccines are usually loaded by NPs, the biocompatibility of TCM vaccines mainly depends on the nanocarrier. The biosafety of NPs is affected by their physicochemical properties, such as size, structure, shape, aggregation state, surface chemistry, and stability.^[^ ^302^ ^]^ Compared with other types of modified NPs, NPs with TCM coating provide some advantages: (1) Did not change the size of NPs significantly. Different cells prefer to take NPs with distinct sizes, for example, DCs usually ingest virus‐like particles (20–200 nm)^[^ ^303^ ^]^ while macrophages prefer to engulf larger ones (0.5–5 µm).^[^ ^304^ ^]^ As novel vaccines, TCM coating can maintain DC uptake efficiency by controlling the size of NPs. (2) TCM provides a larger platform for surface engineering and serves as a barrier to some toxic metal ions, such as Ag^+^. (3) TCM changes the charge of NPs which effectively reduces uptake by non‐immune cells. Besides, TCM naturally targets tumor tissue, which elevates the efficiency of APC uptake in tumor microenvironment. (4) TCM provides tumor antigens to trigger anti‐tumor immunity, to a relatively stronger degree then against NPs. Compared with TCM wrapping NPs, TEVs supply DAMPs and intracellular tumor antigens additionally that triggers innate immunity and expand the spectrum of anti‐tumor immunity.

Despite their efficacy in animal models, TCM vaccines have yet to be applied clinically. The irradiated whole autologous tumor cell vaccines all failed to significantly prolong the median OS of patients with different cancers.^[^ ^23^ ^]^ One of the possible reasons why the vaccines failed is that the abundance in the regulatory factors in immunology and metabolism remodels TMEs and impedes anti‐tumor immunity, a plausibly more critical reason than patient heterogeneity and study design flaws. As the repertoire of molecules of tumor information transfer, TCMs are rich in factors of immune escape. A large number of researches have revealed that TEVs can inhibit T cell activation via PD‐L1, IL‐10, prostaglandin E2 and Fas‐L, and promote Treg proliferation by activating TGF‐β/SMAD pathway. TEVs also carry NKG2D and TGF‐β which hinder the cytotoxicity of NK cells and drive M2 macrophage polarization.^[^ ^305^, ^306^ ^]^ Apart from immunity interference, TMVs have been revealed to accelerate tumor angiogenesis via the transfer of tumor cargoes to endothelial cells.^[^ ^307^ ^]^ These revelations hinder the application of TEV vaccines in the clinic, with TEXs and TMVs from successful clinical trials in cancer therapies only serving as adequate carriers and innate immune stimulators instead of vaccines.^[^ ^308^ ^]^ Originating from normal cells, tumor cells contain many antigens common to normal cells, raising the risk of the mobilization of CTL responses to normal tissues by the stimulation of TCM and its derivatives. To overcome these obstacles, the mechanisms of TCM promoting cancer progression need further elucidation, which sets the foundation for the use of agents that neutralize these factors.

## 6 CONCLUSION

In the review, we have provided the development of TCM vaccines and their derivatives. With tumor antigens, TCM can combine with different NPs, like polymeric, inorganic NPs, and viral particles to protect and treat tumors by igniting specific immune responses against tumor. TCMNPs can serve as carriers of agents of chemotherapy, sonodynamic therapy, phototherapy, and oncolytic viruses. With modification of co‐stimulatory molecules, inhibitors of immunosuppressive and novel adjuvants like Mn, TCM vaccines further activate innate and adaptive immunity to eliminate cancer cells. Besides TCM vaccines, TEVs additionally provide intracellular antigens of tumor with better biocompatibility. Through stimulation inducing stress and damage like mechanical extrusion, UV, radiation, and MWA, TEVs can produce more DAMPs that trigger the innate immune pathway of APCs when they are captured by APCs, resulting in inhibition of tumor progression. The development of tumor membrane‐based vaccines is still in its infancy. Further efforts in fusion methods, functional modification, industrial manufacturing, novel RCD induction, and identification of neoantigens should provide bases for improving the clinical landscape of cancer treatment.

## CONFLICT OF INTEREST STATEMENT

The authors declare no conflicts of interest.
